# A deep convolutional neural network for classification of red blood cells in sickle cell anemia

**DOI:** 10.1371/journal.pcbi.1005746

**Published:** 2017-10-19

**Authors:** Mengjia Xu, Dimitrios P. Papageorgiou, Sabia Z. Abidi, Ming Dao, Hong Zhao, George Em Karniadakis

**Affiliations:** 1 Key Laboratory of Medical Image Computing of Ministry of Education, Northeastern University, Shenyang, China; 2 Division of Applied Mathematics, Brown University, Providence, Rhode Island, United States of America; 3 Department of Materials Science and Engineering, Massachusetts Institute of Technology, Cambridge, Massachusetts, United States of America; University of California Irvine, UNITED STATES

## Abstract

Sickle cell disease (SCD) is a hematological disorder leading to blood vessel occlusion accompanied by painful episodes and even death. Red blood cells (RBCs) of SCD patients have diverse shapes that reveal important biomechanical and bio-rheological characteristics, *e.g.* their density, fragility, adhesive properties, etc. Hence, having an objective and effective way of RBC shape quantification and classification will lead to better insights and eventual better prognosis of the disease. To this end, we have developed an automated, high-throughput, ex-vivo RBC shape classification framework that consists of three stages. First, we present an automatic hierarchical RBC extraction method to detect the RBC region (ROI) from the background, and then separate touching RBCs in the ROI images by applying an improved random walk method based on automatic seed generation. Second, we apply a mask-based RBC patch-size normalization method to normalize the variant size of segmented single RBC patches into uniform size. Third, we employ deep convolutional neural networks (CNNs) to realize RBC classification; the alternating convolution and pooling operations can deal with non-linear and complex patterns. Furthermore, we investigate the specific shape factor quantification for the classified RBC image data in order to develop a general multiscale shape analysis. We perform several experiments on raw microscopy image datasets from 8 SCD patients (over 7,000 single RBC images) through a 5-fold cross validation method both for oxygenated and deoxygenated RBCs. We demonstrate that the proposed framework can successfully classify sickle shape RBCs in an automated manner with high accuracy, and we also provide the corresponding shape factor analysis, which can be used synergistically with the CNN analysis for more robust predictions. Moreover, the trained deep CNN exhibits good performance even for a deoxygenated dataset and distinguishes the subtle differences in texture alteration inside the oxygenated and deoxygenated RBCs.

## Author summary

There are many hematological disorders in the human circulation involving significant alteration of the shape and size of red blood cells (RBCs), e.g. sickle cell disease (SCD), spherocytosis, diabetes, HIV, etc. These morphological alterations reflect subtle multiscale processes taking place at the protein level and affecting the cell shape, its size, and rigidity. In SCD, in particular, there are multiple shape types in addition to the sickle shape, directly related to the sickle hemoglobin polymerization inside the RBC, which is induced by hypoxic conditions, e.g., in the post-capillary regions, in the spleen, etc. Moreover, the induced stiffness of RBCs depends on the de-oxygenation level encountered in hypoxic environments. Here, we develop a new computational framework based on deep convolutional networks in order to classify efficiently the heterogeneous shapes encountered in the sickle blood, and we complement our method with an independent shape factor analysis. This dual approach provides robust predictions and can be potentially used to assess the severity of SCD. The method is general and can be adapted to other hematological disorders as well as to screen diseased cells from healthy ones for different diseases.

## Introduction

Sickle cell disease (SCD), also known as sickle cell anemia, is a type of inherited RBC disorder associated with abnormal hemoglobin S (HbS) [1]. When HbS molecules polymerize inside RBCs, due to lack of oxygen, they affect greatly the shape, elasticity, and adhesion properties of RBCs. Moreover, the RBCs become stiff and more fragile, with vastly heterogeneous shapes in the cell population [2], which makes this problem an ideal candidate for the examination of morphological heterogeneity. Unlike the normal RBCs, which are flexible and move easily even through very small blood vessels, sickle RBCs promote vaso-occlusion phenomena. Hence, SCD patients are afflicted with the risk of life-threatening complications, stroke and organ damage over time, resulting in a reduced life expectancy. According to a recent study [3], as of 2013 about 3.2 million people have SCD while an additional 43 million have sickle-cell trait, resulting in 176,000 deaths in 2013, up from 113,000 deaths in 1990, mostly of African origin. The prime hallmark of SCD is that is surprisingly variable in its clinical severity. Available methods for treating SCD are mainly supportive and mostly aim at symptom control, but lack the active monitoring of the health status as well as the prediction of disease development in different clinical stages [4]. Recent developments in advanced medical imaging technology and computerized image processing methods could provide an effective tool in monitoring the status of SCD patients. Indeed, Darrow et al. [5] recently demonstrated a positive correlation between cell volume and protrusion number using soft X-ray tomography. Van beers et al. [6] have also shown highly specific and sensitive sickle and normal erythrocyte classification based on sickle imaging flow cytometry assay, a methodology that could be useful in assessing drug efficacy in SCD.

Therefore, implementing an automated, high-throughput cell classification method could become an enabling technology to improve the future clinical diagnosis, prediction of treatment outcome, and especially therapy planning. However, there are several major technical challenges for automatic cell classification: 1) RBCs may touch or overlap each other or appear as clusters in the image, which makes it difficult to detect the hidden edge of cells. 2) The RBC region and the background may have low contrast in the intensity. 3) The boundaries of RBCs may be blurry due to the influence of imaging procedure. 4) Very complex and heterogeneous shapes of RBCs are present in SCD. 5) Artifacts may be present, for instance, dirt on the imaging light path, various halos and shading. 6) Finally, because RBCs lack a nucleus, methods utilizing the nuclei location as an apparent marker for cell counting and detection are not applicable.

The objective of the current work is to develop an automated algorithm for sickle RBC classification test, which may prove a powerful complementary clinical test for a) assessing patient’s disease severity via longitudinal tracking and patient-specific RBC mapping, and b) intervention strategies via personalized medicine treatment monitoring. Next, we present a brief overview of the state-of-the-art techniques involved in cell segmentation and classification.

Cell detection methods are prevalent, see e.g. [7–10], and some open source software (e.g., CellProfiler [11], CellTrack [12], Fiji [13] and CellSegm [14], etc.) for 2D and 3D cell detection and counting has emerged recently. However, in SCD we need cell classification, which is quite difficult due to the heterogeneous shapes of RBCs and the existence of touching and overlapped RBCs in the raw microscopy image, and existing software cannot be directly used to obtain RBC boundaries and cannot distinguish among the many different types of RBCs. Presently, there are two kinds of cell classification approaches, i.e., manual and automatic. In the manual approach one inspects the blood samples using the microscope to count the number of cells and examines the outliers in each frame. This, apparently, is subjective, labor intensive and time consuming for batch data processing. Coulter Counters and Laser Flow Cytometers enable cell sorting automatically by detecting the current and light refraction changes during cell pass through the channel. However, there are some shortcomings, such as the high cost and low processing speed (106 cells/hour), and in particular, these instruments are not suitable for the classification of heterogeneous cells. Thus, some cellular data analysis tools have been recently developed targeting this problem. For example, ACCENSE [15] adopted two clustering methods (k-means and DBSCAN) to facilitate the cellular classification automatically, however, the clustering performance relies on the properly initiation of parameters by hand; moreover, the performance of cell classification degrades for the clusters with different size and different density. More recently, RSIP Vision (http://www.rsipvision.com) has developed a commercial software package, allowing the recognition and count of RBCs by using a classifier to classify the hand-crafted morphological features; however, the main drawback of this method is that it requires domain-specific expertise on the feature extraction,f and is also a time-consuming procedure. In addition, the accuracy of the method has not yet been demonstrated for cell classification. Both of the aforementioned methods use machine learning tools but not deep learning algorithms. Likewise, some other similar studies on the HEp-2 cell classification based on the traditional machine learning methods have emerged recently, such as in [16] where multi-variant linear descriptors were adopted to extract the features and applied the SVM method to realize HEp-2 cell classification with an accuracy of 66.6%. Other methods include superpixels-based sparse coding method approach [17], k-nearest clustering method for red blood cell and white blood cell classification [18], etc.

Due to ineffectiveness of the aforementioned methods and given the recent advances of deep learning technique, Gao et al. [19] performed HEp-2 cell classification based on deep CNNs. Also, in order to improve the diversity of single HEp-2 cell data samples, Li et al. [20] carried out classification experiments based on deep CNNs by using four different patients’ datasets under different lighting conditions. However, for the currently available automated machine learning methods, which could be used for cell classification, the following are still drawbacks: 1) the classification studies are mostly directly based on already prepared single HEp-2 cellular data, hence, ignoring the initial key procedure of single cell extraction from the raw image data; 2) the adopted conventional machine learning methods are time consuming for the hand-crafted feature extraction and need specific human expertise; moreover, they need an accurate cell segmentation; 3) the classification accuracy is limited by the selected features and the performance of selected classifier. For our application, since RBCs of SCD exhibit special characteristics in terms of heterogeneous shapes and variant sizes, there is still no efficient tool that can be used to facilitate the automated inspection and recognition of various kinds of RBC patterns which are present in SCD blood.

The main focus of our paper is to develop an automated, high-throughput sickle cell classification method based on the deep Convolutional Neural Networks (dCNNs), taking advantage of the hierarchical feature learning goodness of dCNNs. The rest of this paper is organized as follows: In Section 3 we present our methodology, and in Section 4 we present the experimental results, a comparative analysis, and a discussion. Finally, in Section 5 we present the conclusion. S1–S4 Appendix contain some more details of the collection of raw data, the shape factor analysis, the CNN architecture, and deoxygenation method of sickle RBCs.

## Materials and methods

**Fig 1:**
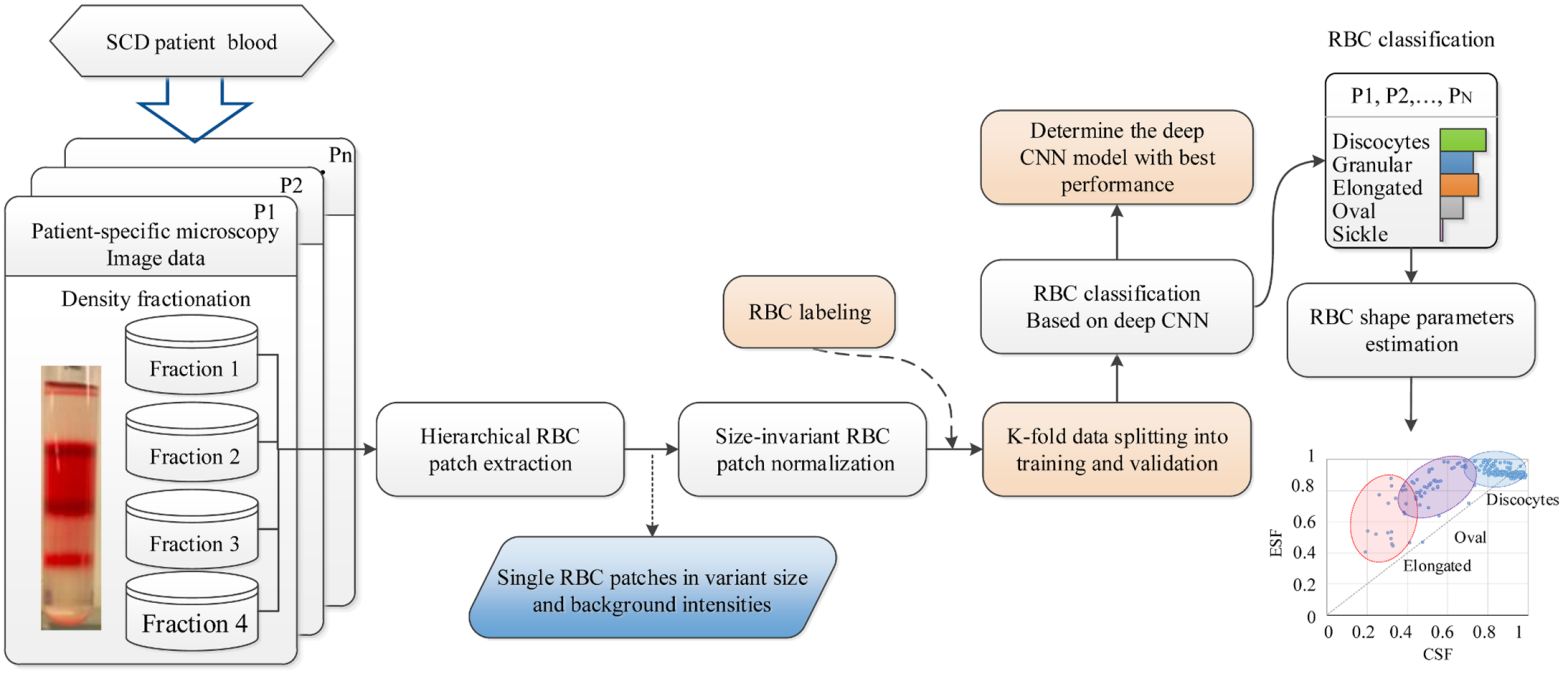
Overall flowchart of our proposed training and learning methodology for the sickle RBC-dCNN classification model describing the four main steps, including an independent shape factor analysis.

On the basis of the raw RBC microscopy image data from SCD patients following cell density fractionation [21] as shown in S1 Appendix, our automatic, high-throughput RBC classification assay consists of four main steps for the RBC-dCNN training: 1) Hierarchical RBC patch extraction, 2) Size-invariant RBC patch normalization, 3) RBC pattern classification based on deep CNN, and 4) Automated RBC shape factor calculation. A detailed overall training flowchart is shown in Fig 1. Each step of the algorithm is described below.

### Hierarchical RBC patch extraction and size-invariant RBC patch normalization

In the traditional learning-based cell image segmentation or classification method, the two most common techniques to obtain the training patches are the exhaustive pixel-wise sliding window with the same size method [22] and the ground truth bounding box method, *e.g.* Li et al. [20]. However, the major drawback of the pixel-wise block splitting method for the application of RBC classification is that it generates a large number of unwanted and redundant patches for the background and artifacts (*e.g.*, dirt or debris in the light path) to feed for training and testing of the neural network. This redundancy and artifacts significantly hinder the efficiency of the method to take into account the high resolution of the microscopy data and large background area. The ground truth bounding box method was based on manual labeling of cells present in raw images, a process which is labor intensive and needs specific domain knowledge.

**Fig 2:**
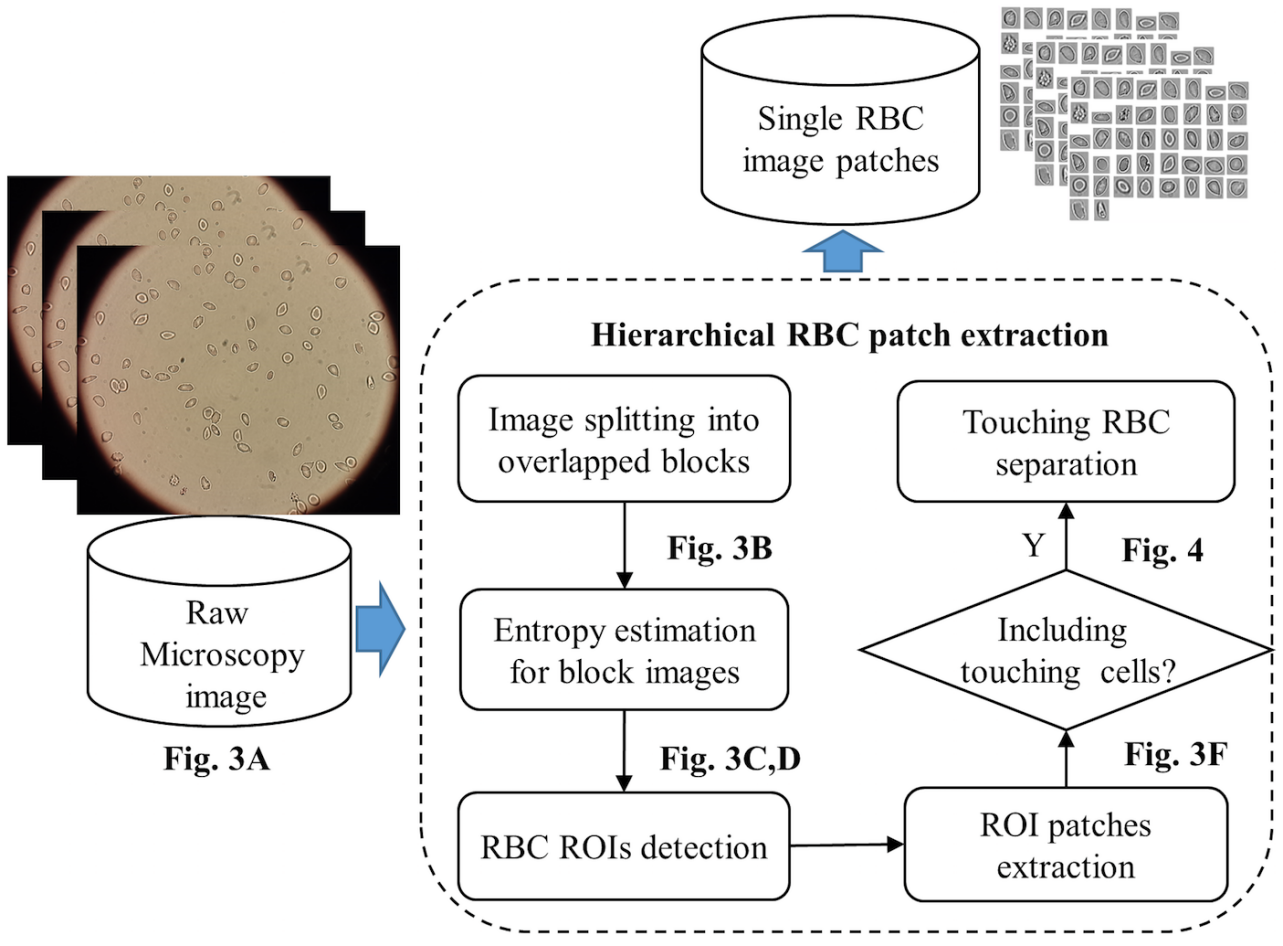
Hierarchical RBC patch extraction algorithm workflow. See also Figs 3 and 4 for details of each step.

In addition, due to the fact that sickle cells are always heterogeneous in shape and at times touch or overlap, it can be difficult to obtain all single RBC patches by using the sliding or bounding box window with a fixed pixel size. Therefore, in our study, a hierarchical RBC patch extraction method was developed to overcome the above problems. The complete flowchart of the proposed method is shown in Fig 2.

Firstly, the raw microscopy images were divided into overlapped patches by using the sliding window technique, with the block size *N* * *N*. Then, the entropy containing in each image block was estimated by Eq (1) below:

$$\begin{array}{c} E=-\sum_{i=1}^{L}P_{i}\log P_{i}, \end{array}$$ (1)

where *L* is the maximum grayscale level, *P*_*i*_ refers to the probability of occurrence for each intensity level that is encountered in the image block, and it can be derived from the *i*th histogram count *f*(*i*, *j*) divided by the amount of pixels in each subblock image (the size of the block is *N*), as shown in Eq (2) below:

$$\begin{array}{c} P_{i}=\frac{f(i,j)}{N^{2}}. \end{array}$$ (2)

**Fig 3:**
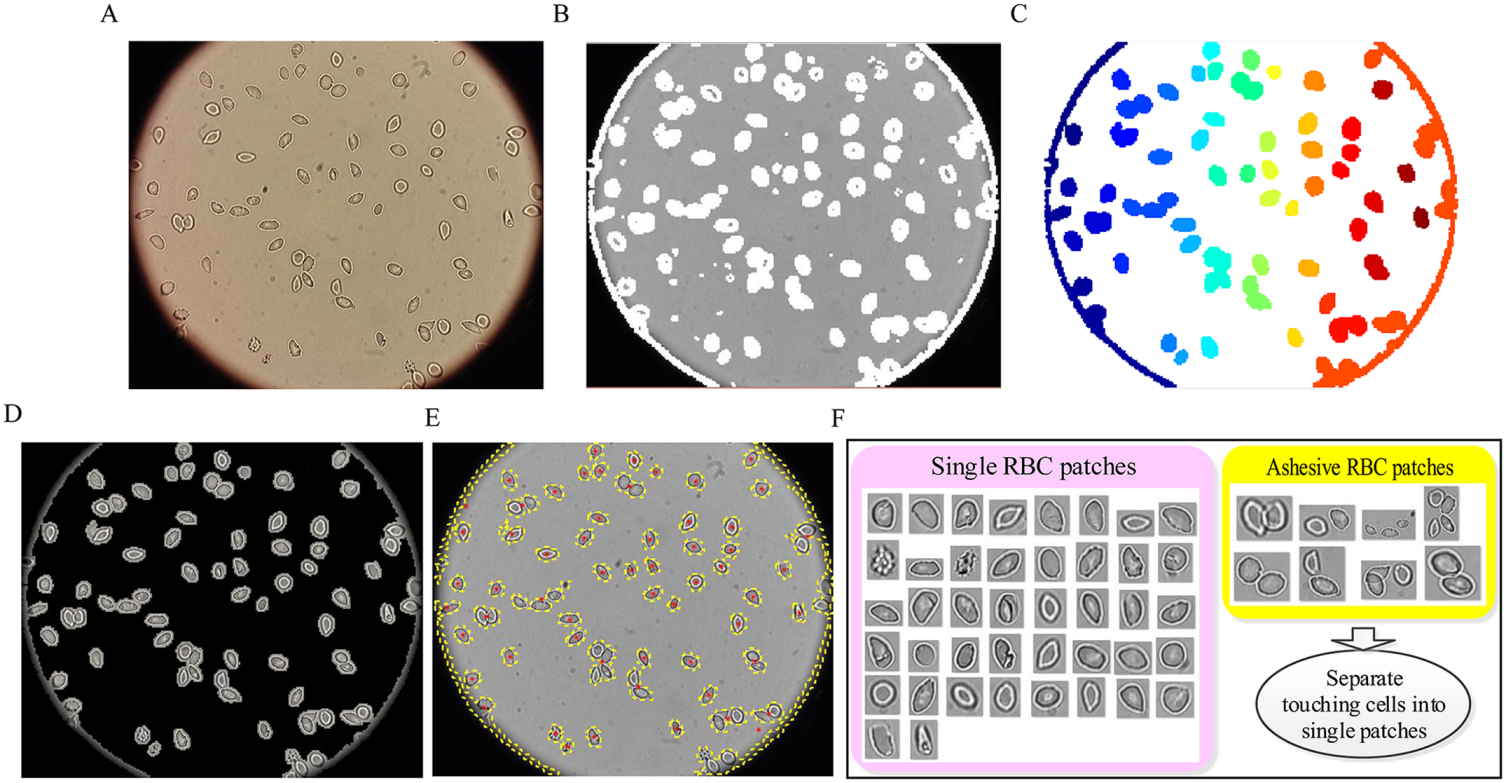
Determining ROIs and RBC patch extraction based on information from the entropy statistical estimation and morphology operations. (A) raw microscopy image. (B) Blocks with high entropy. (C) ROI mask image. (D) Detection of ROIs. (E) Boundaries of ROIs. (F) Single & “touching/overlapped”.

We have employed the information entropy to measure the uncertainty in RBC regions and the background region; the high entropy regions were extracted as the ROI (region of interest), *i.e.* the RBC regions in the raw microscopy images. The detailed ROI extraction procedure is shown in Fig 3.

First, raw microscopy images (Fig 3A in high resolution were split into overlapped blocks. Next, the information entropy was calculated for all sub-blocks (including the edges and noises blocks). The blocks with high entropy are shown in white color in Fig 3B, where the entropy threshold (5.0) was obtained from our validation experiments on different datasets. The corresponding ROI mask image was generated by filling the holes and removing the artifacts with area smaller than a common RBC prior area (6*10*10) for the result of Fig 3B. The result is shown in Fig 3C with each color representing a single ROI region. Fig 3D shows the “cleaned” RBC ROI region result corresponding to the ROI mask image. The entropy estimation method can effectively extract the complete RBC regions from the raw images, especially for those RBC boundaries in a low intensity contrast. Moreover, it can also detect the RBC region correctly from various datasets regradless of their brightness differences. Thus, it can effectively overcome the shortcomings of the previous commonly used methods (*e.g.*, Ostu, watershed and Sobel, etc.). To obtain the RBC patch images for the deep CNNs, the high-level ROI boundary is detected and by searching the minimum coordination of pixel (*x*_0_, *y*_0_) and maximum pixel coordination (*x*′, *y*′) from the boundary pixels, the ROI patches are illustrated as shown in Fig 3E.

**Fig 4:**
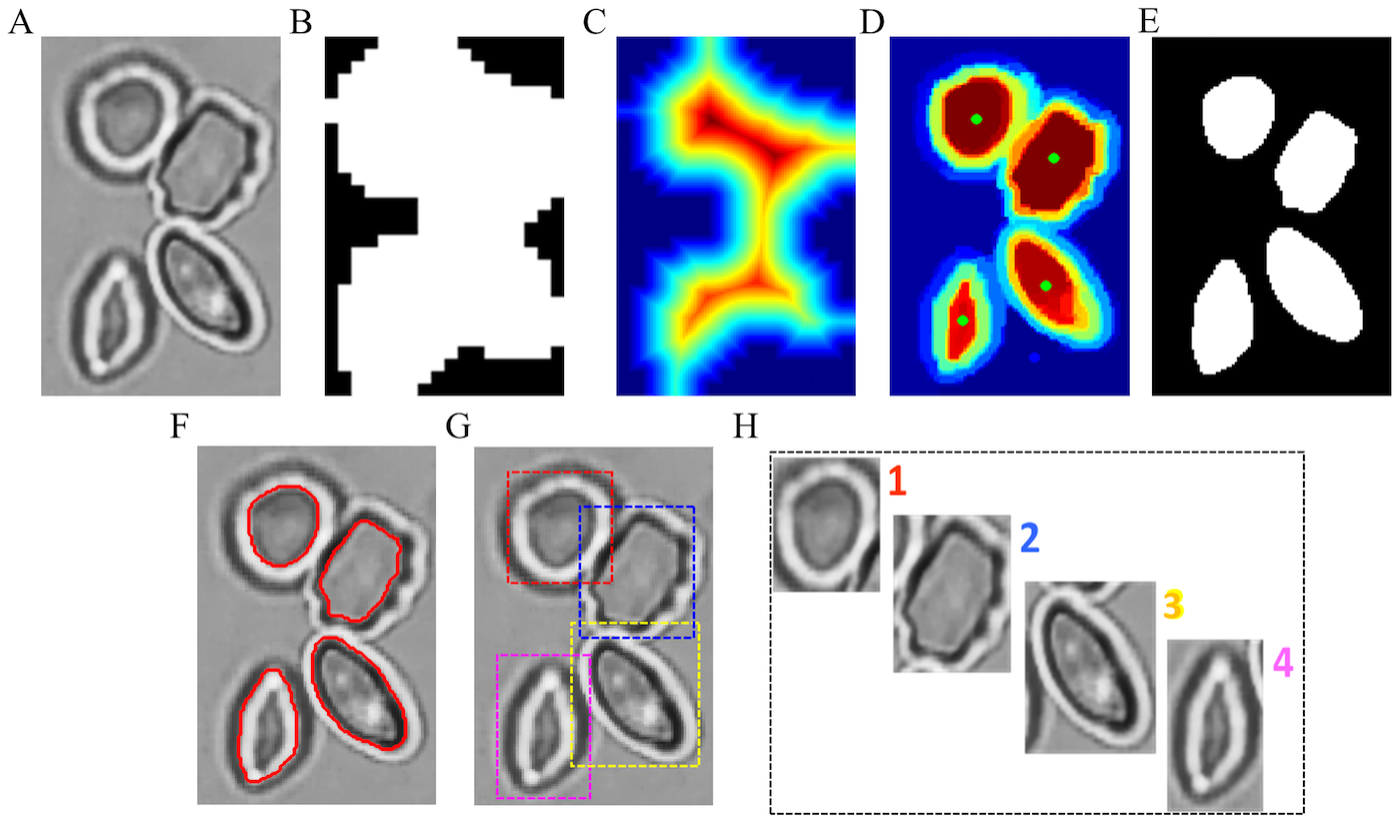
Workflow of RBC patch generation from ROIs with touching RBCs. (A) ROI patch. (B) binary ROI mask image. (C) Euclidean distance transfrom result. (D) Probability map based on random walk method with seeds (green dots) obtained from distance transform result. (E) separated RBC binary mask. (F) segmented RBC outlines (red line). (G) bounding boxes of single RBCs. (H) The touching RBCs are separated into four single RBC patches.

It should be noted, however, that for the particular situation of overlapped and touching RBCs that may be present in the raw microscopy image, we may obtain some extracted ROI regions containing multiple cells; see the yellow smaller sized box in Fig 3F, where 8 ROI patches contain two or more RBCs, and the pink smaller sized box that includes all segmented single RBC patches. The subimages in the two boxes were obtained by calculating the corresponding bounding boxes of the ROI. Overlapping RBCs were removed from the input of deep CNNs in our work. Therefore, we only focused on the “touching” RBC separation problem by applying the random walk method [23] in conjunction with the distance transform [24] to generate the RBC boundary. This method can obtain the RBC seed points identification automatically. The specific separation procedure is shown in Fig 4.

**Fig 5:**
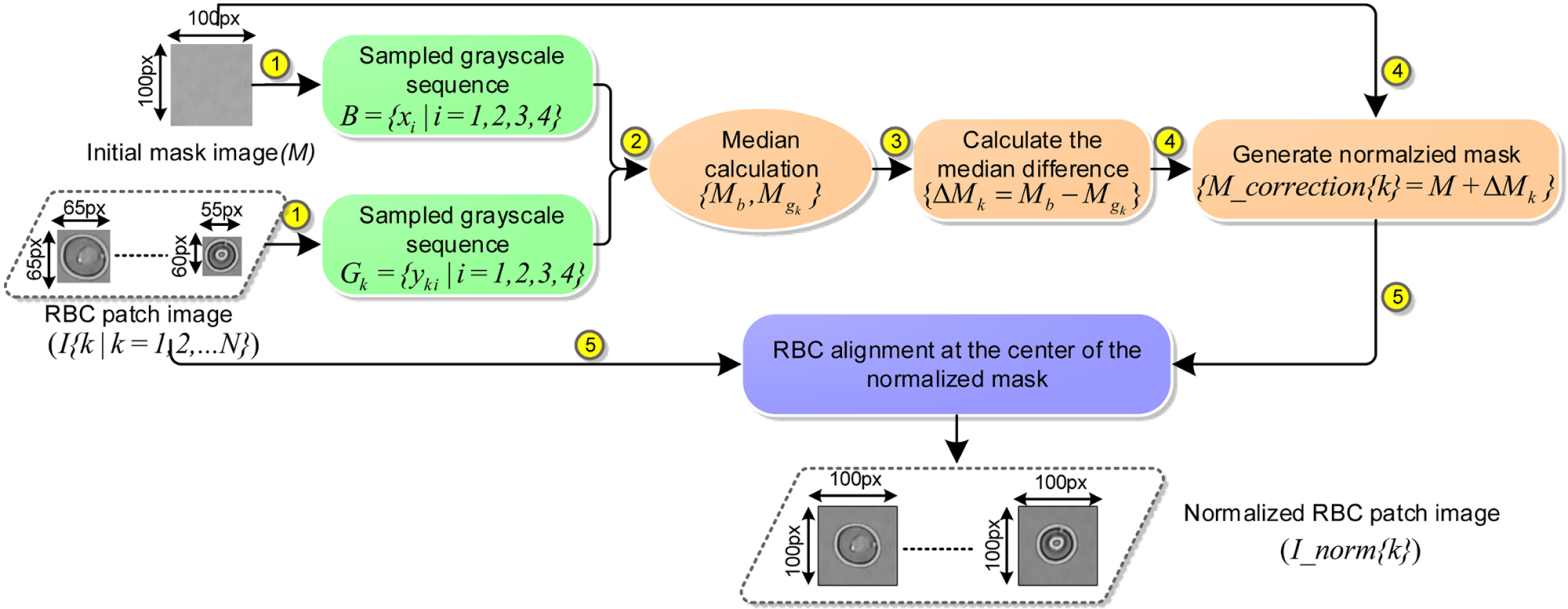
Workflow of the size-invariant (100px*100 px) RBC patch normalization. Steps 1-5 are described in the text.

Because of the RBC heterogenity in size, shape and orientation, the generated single RBC patches from section B were of different sizes (see Fig 3E). In addition, due to varying brightness and intensity contrast conditions during the procedure of raw RBC microscopy data collection, the background of RBC patch images appeared to differ among datasets. Currently, commonly used image scaling methods for the image size normalization are prone to reducing the RBC patch image fidelity (*e.g.*, intensity contrast, noise and distortion), which will accordingly affect the RBC classification accuracy of the CNN. Therefore, to overcome the above issues, a size-invariant RBC patch normalization method based on statistic intensity linear mapping was employed. The algorithmic workflow is shown in Fig 5.

**Fig 6:**
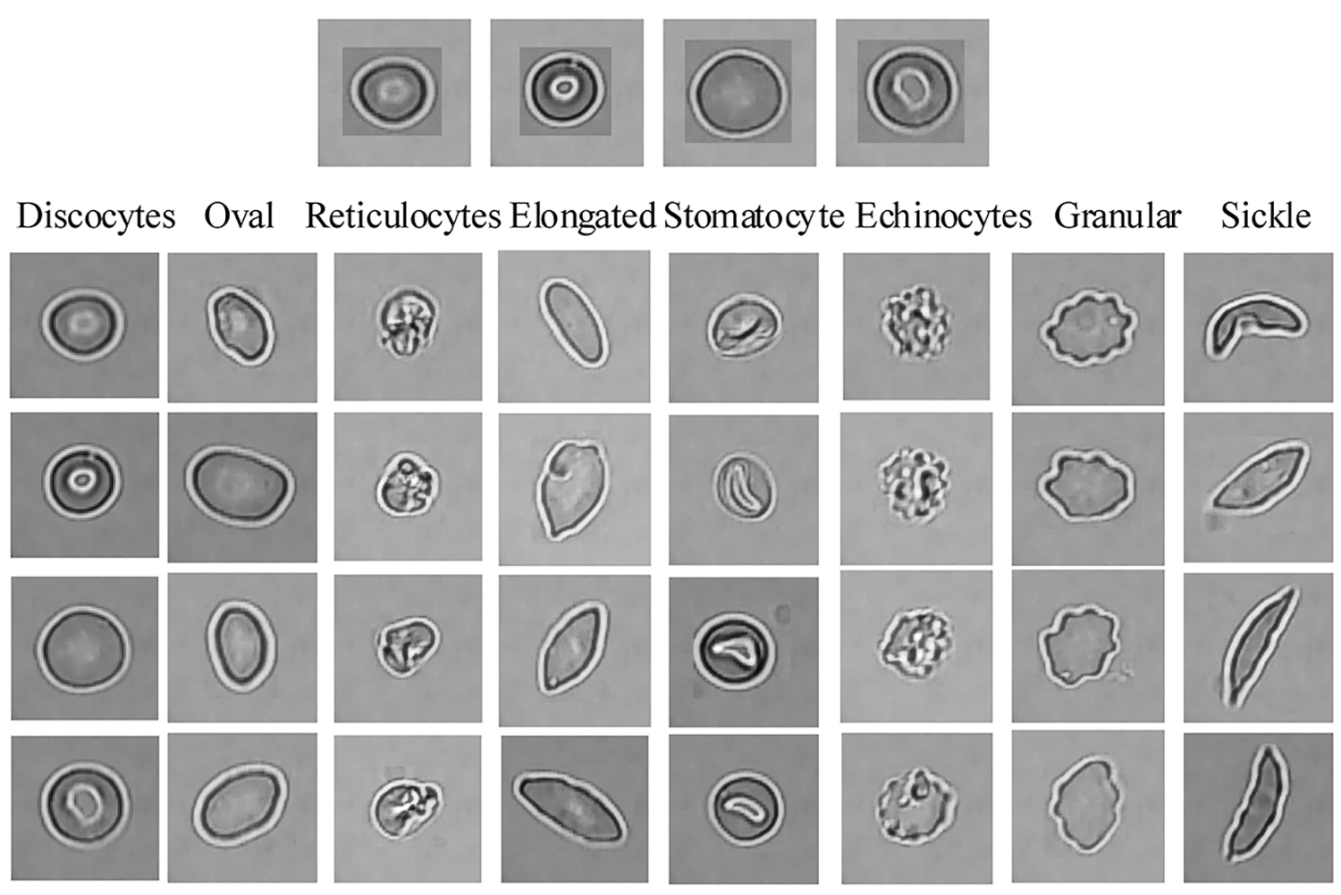
Size-invariant RBC patch size normalization for eight different types of diseased RBCs (horizontal) and four different groups (vertical). Images in the first row should be compared against the first column of “Discocytes”.

1. **Initial normalization mask selection.** A background region with size of 100*100 pixels was cropped from the raw microscopy image as the initialized normalization mask, as shown in ① of Fig 5.
2. **Adaptive normalization mask generation based on statistical intensity linear mapping.** Because the background area of every microscopy image as well as the single RBC patches have a uniform intensity distribution and a low mean intensity difference, we utilized a linear intensity mapping method (see steps from ② to ④ in Fig 5) to estimate the intensity difference between the unnormalized RBC patch and the standard normalization mask. Firstly, *G*_*k*_ = {*y*_*ki*_|*i* = 1, 2, 3, 4} refers to the intensity sequence sampled from the *k*th RBC patch image (*I*), and *B* = {*x*_*i*_|*i* = 1, 2, 3, 4} is the intensity sequence of initial standard normalization mask (*M*); *x*_*i*_, *y*_*i*_ are the intensity values corresponding to the 4 sampled corners in the mask and patch image. In order to avoid extremely large or small grayscale values, we also employed the statistical median of *G*_*k*_ and *B* to estimate the background intensity difference (Δ*M*) between patch image *I* and the standard mask *M*. The median calculation is given in Eq (3) below.
  $$\begin{array}{c} M_{G}=\{\begin{array}{cc} x_{(N+1)/2}, & \text{if}\;N\;\text{is}\;\text{odd,}\\ (x_{N/2}+x_{N/2+1})/2, & \text{if}\;N\;\text{is}\;\text{even.} \end{array} \end{array}$$ (3)
  Here, *G*{*x*_*i*_|*i* = 1, 2, …, *N*} is an arranged sequence, and *N* is the total number of observations. Finally, an adaptive normalization mask image was generated by performing the linear intensity mapping technique on the initial normalization background mask *M* according to the calculated intensity difference (Δ*M*); the details are shown in ④ of Fig 5.
3. **Align normalization mask with corresponding RBC patch.** All single RBC patches in their respective various sizes were aligned at the center of their corresponding normalization mask, see ⑤ in Fig 5. In order to validate the performance of our method, we performed the proposed method on multiple RBC patches from different datasets with different background intensity distribution. Examples of the normalised size-invariant RBC patches from different datasets in Fig 6 show that the RBC shape remains unaltered and staircase artifact free during the algorithmic operations.

### RBC pattern classification based on deep CNN

In our work, we adopted a deep CNN architecture with 10 layers, including 3 convolutional layers (*C*1, *C*3 and *C*5), 3 pooling/subsampling layers (*P*2, *P*4 and *P*6), dropout layers (*D*7 and *D*9, where *p* = 0.5) and a fully connected layer (*F*8). As a result of the computational efficiency, the grayscale RBC image patches were initially resized to 78 * 78. Next, these were then fed into the neural network. A ReLU non-linear activation function was then applied. Following the *F*7 layer, a logistic regression method combining the softmax function (see Eq (4)) with a cross-entropy loss function (see Eq (5)) was implemented to obtain the final learning probability and predicted labels. The softmax function can “squash” the obtained score vector *Q* = {*q*_*i*_|*i* = 1, 2, …, *N*} to a N-dimension probability vector *δ*(*q*_*i*_), so as to aid RBC classification efficiency.

$$\begin{array}{cc} & Q^{\prime }=\delta \left(q_{i}\right)=e^{q_{i}}/\sum_{j=1}^{N}e^{q_{j}}, \end{array}$$ (4)

$$\begin{array}{cc} & D\left(Q^{\prime },Q\right)=-\sum_{i=1}^{N}Q_{i}\log\left(Q_{i}^{\prime }\right), \end{array}$$ (5)

**Fig 7:**
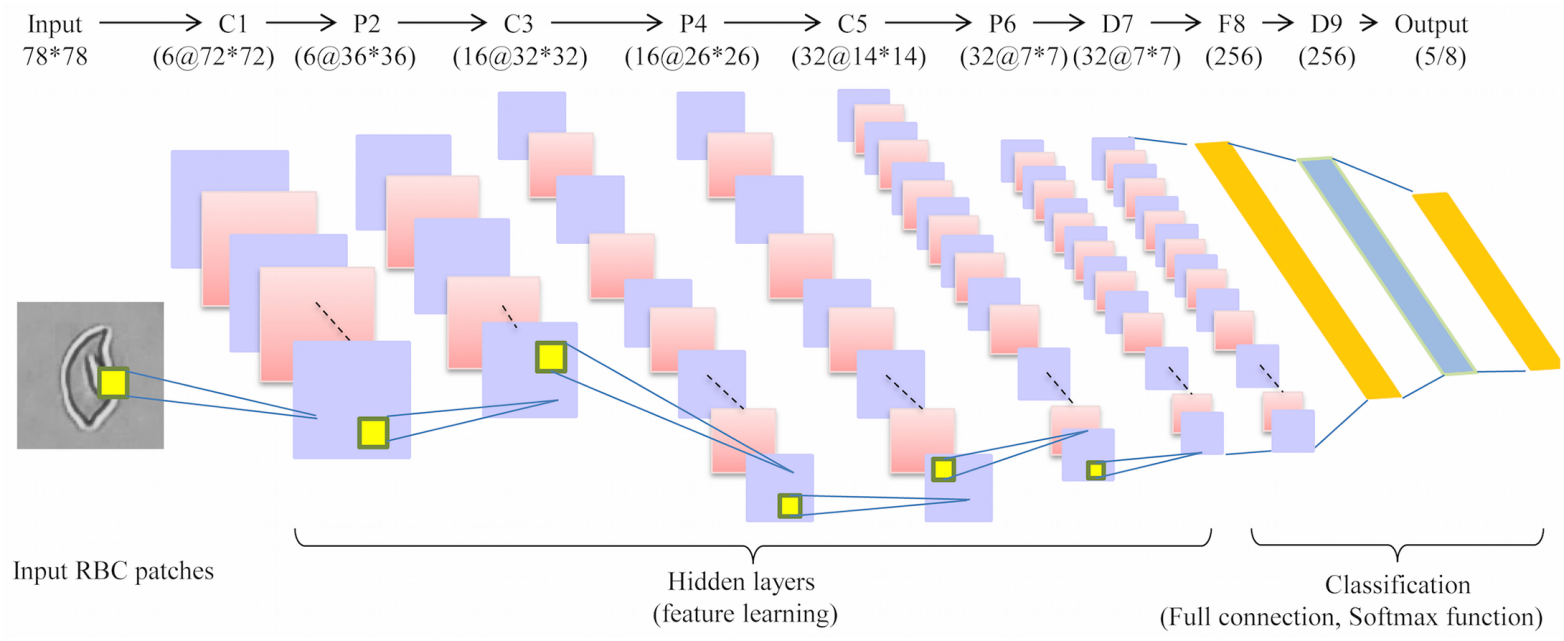
Architecture of deep RBC-dCNN for SCD RBC classification.

According to different shape division level for the original RBC patches, two kinds of RBC labeling principles were employed in the experiment: *coarse labeling*(output = 5) and *refined labeling* (output = 8). Thus, the output layer had two different dimensions (5 or 8 categories). More details about the deep CNN architecture applied in this paper are shown in Fig 7 (see also S3 Appendix for the specific illustration of the layers of deep CNN).

### Automated RBC shape factor analysis following classification

As mentioned previously in the text, RBCs from SCD patients vary significantly in morphology/shape [25]. In the previous section, deep CNNs was applied to train and learn the diverse RBC patterns from RBC microscopy imaging data (see Table 1). Hence, by utilizing this deep CNNs we can classify sickle RBC in different types according to training. In addition to RBC type classification we perform shape factor analysis for each RBC type to further quantify specific RBC shape parameters derived from the contour analysis of the individual RBCs. Three kinds of shape factors were calculated in this work.The shape factors’ formulas and pseudo-code for the specific implementation of the automatic RBC shape factors quantification method are given in S2 Appendix.

- **Regional shape factors:** Segmented area and perimeter of RBC (*Area*_*r*, *Perm*_*r*), convex area and perimeter (*Area*_*c* and *Perm*_*c*), maximum Feret diameter (*maxFD*), minimum Feret diameter (*minFD*).
- **Elliptical shape factors:** Short axis (*Rb*), Long axis (*Ra*), Rotation angle (*θ*), etc.
- **Derived shape factors:** Ellipticity shape factor (*ESF*), Circular shape factor (*CSF*), Elongation, Convexity (*Conv*) and Compactness (*Compt*).

**Table 1:** Description of our experimental dataset from eight patients’ imaging data.

No.	RBCs Type	Dataset_1 (4 patients)	Dataset_2 (3 more patients)	Total number of RBCs	Exp_I (4 patients)	Exp_II (8 patients)
1	Discocytes[Dic]	338	-	338	2028	2028
2	Echinocytes[Ech]	89	34	123	534	738
3	Elongated[El]	280	-	280	1680	1680
4	Granular[Grl]	75	16	91	450	546
5	Oval[Ovl]	118	-	118	708	708
6	Reticulocytes[Ret]	82	33	115	492	690
7	Sickle[Sk]	22	93	115	132	690
8	Stomatocytes[Sto]	24	-	24	144	144
**Total number of RBCs**		1030	176	1204	6168	7224

On the basis of the above automated image-based shape factor analysis scheme, we can perform a comprehensive shape analysis for the classified RBCs or unclassified RBCs according to specific practical applications and requirements.

## Results/discussion

**Fig 8:**
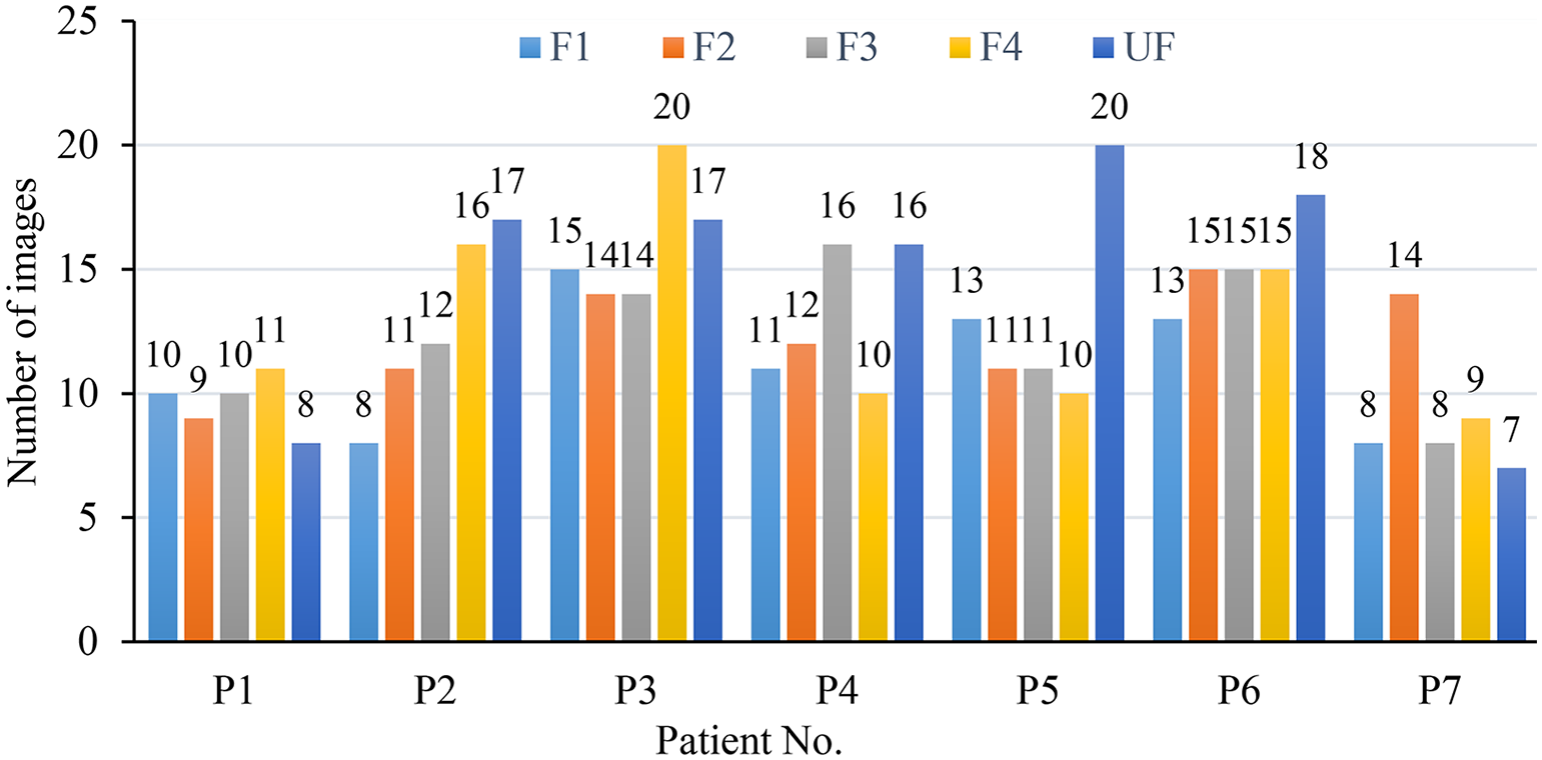
Distribution of the number of images in five fractions corresponding to seven different patients.

In this section, we conduct several experiments to evaluate the performance of the deep CNN used in the special RBC classification cases and present a comparative analysis of the results. In our experiments in order to validate the robustness of our methodology in dealing with different imaging data, we consider 434 raw microscopy images of 8 different SCD patients collected from two different hospitals. The number of images for each patient in different fractions is shown in Fig 8; all the images in different fractions (F1,F2,F3,F4 and UF) are of the same size (1920*1080 pixels) in TIFF format with 4 color channels. Based on the obtained raw images, 7206 single RBC image patches were extracted by using the proposed method in Section 3.1.Subsequently, all RBC patch images were normalized to the same size (78*78) by using the method described in section 3.2. Namely, all the RBC patch images were assigned to 8 different categories (discocytes, echinocytes, elongated, granular, oval, reticulocytes, sickle and stomatocyte) manually with the corresponding quantity of each RBC category presented in Table 1 as described in [26]. Conventionally, our definition of echinocytes is equivalent to echinocyte type II and III mentioned in [27]. Echinocyte type I is actually the “granular shape” we mention in this manuscript; moreover, wherever the state of oxygenation is not mentioned it implies “Oxy” state. We note that oval shape refers to the shape of the red cells and is not related to Southeast Asian ovalocytosis. This convention is consistent in our training of the dCNN model. A comparison study on the deep CNNs training model for two datasets with different number of patients’ data was conducted. The input data was enhanced with geometric transformations—a method also known as *data augmentation technique*. This technique adds value to base data by adding information derived from rotation, shifting or mirroring, illumination adjustment, etc., and introduces only a slight distortion to the images but without introducing extra labeling costs. A larger dataset can help evaluate and improve the robustness of RBC classification CNN model as well as restraining the common over-fitting problem. Thus, in our work, five types of data augmentation were performed on the normalized single RBC patch: rotate 90°, 180°, 270° and horizontal and vertical reflection.

### RBC-dCNN model training, optimization and K-fold validation

**Fig 9:**
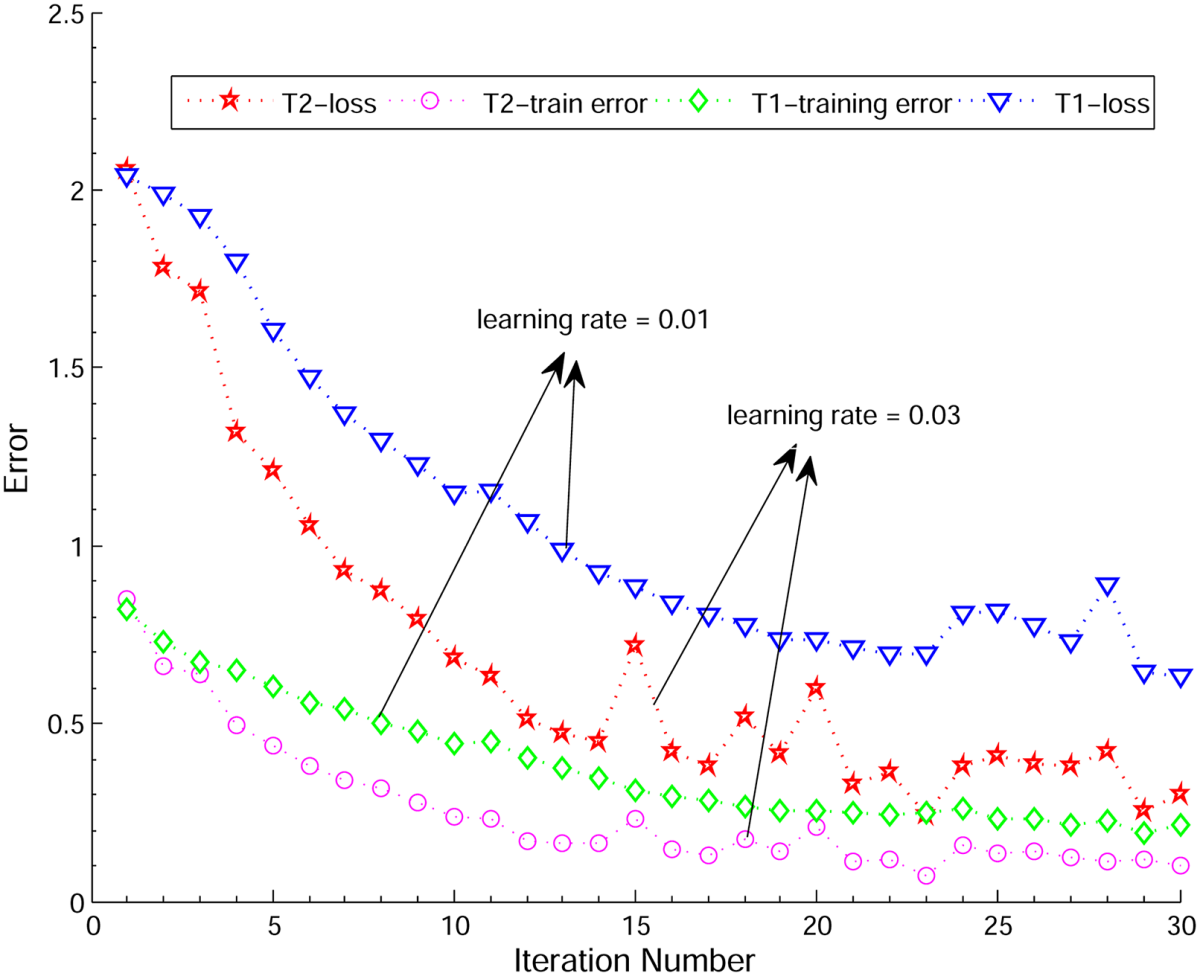
Convergence studies on loss and train error with respect to the number of iterations and different learning rate settings.

**Fig 10:**
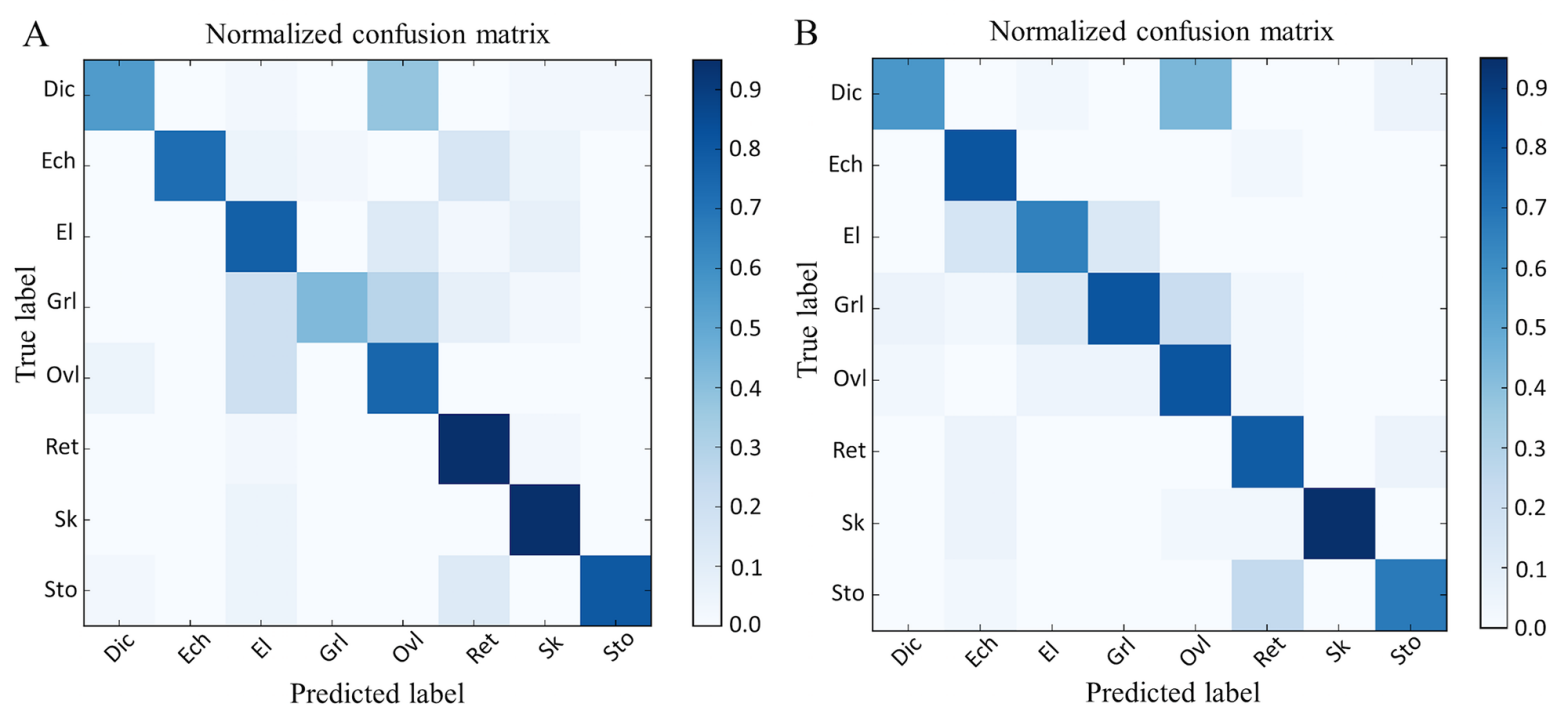
Normalized confusion matrix results with respect to different number of iterations. (A) max_iter = 30. (B) max_iter = 60.

In order to test the performance of the deep convolutional neural network model, we conducted systematic convergence studies with respect to the number of iterations and the learning rate; here we show some representative results. For the case of 4 patients (Exp_I), we evaluated the training error and loss in the configuration of different learning rates (0.01 and 0.03), batch size = 20, image size is 78*78 and weight decay is 0.01. In Fig 9, we observe that both the train and the loss errors decay with the increasing number of epochs, and the higher learning rate can accelerate the decay speed, see the corresponding plots of the loss and train error results for two comparative experiments (*T*1 and *T*2) with different learning rate settings. Moreover, another significant observation in Fig 9 is that both the train error and loss results start fluctuating after 15 iterations for *T*2 and 25 iterations for *T*1. In particular, the fluctuations in the loss increase as the number of iterations increases, but the train error has a relatively smaller fluctuation. In order to better understand the fluctuation problem (so-called “over-training” or “overfitting”), we optimized the batch size and use the *“dropout”* scheme proposed in [28] to overcome this problem. As described before, the dropout layer is implemented after the convolution layer (*p* = 0.5). Finally, when the number of iterations reaches 60, our RBC-dCNN model achieved optimal prediction performance. We plotted the two normalized confusion matrices with respect to different number of maximum iteration times (30 and 60) in Fig 10.

In Fig 10, we observed that the *Discocytes* and *Granular* classes have relative low prediction accuracy among the 8 classes of RBC before the convergence of loss and training error. However, when the maximum number of iterations was 60, there was a significant improvement in the accuracy of different class prediction due to further decay of the loss and training errors. Table 2 gives detailed performance analysis of the running time, train error, test error and loss with respect to different maximum iteration times based on Exp_I dataset.

**Table 2:** Comparisons of loss and train errors based on different iterations.

Iteration	Training error	Loss	Test error	Times(s)
25	0.2094	0.5879	0.3500	2528.4
30	0.1598	0.3867	0.2750	2940.1
40	0.1213	0.3637	0.2469	4011.5
60	0.1026	0.2805	0.2094	5869.6

Despite a learning model being trained to fit the statistics, the model cannot be assumed to have a successful predictive capability. This is due to the regularization which increases the performance, while the performance on test is optimal within a range of values of the regularization parameter. Thus, accurate evaluation of predictive performance is a key step for validating the precision and recall of a deep neural network classification model.

**Fig 11:**
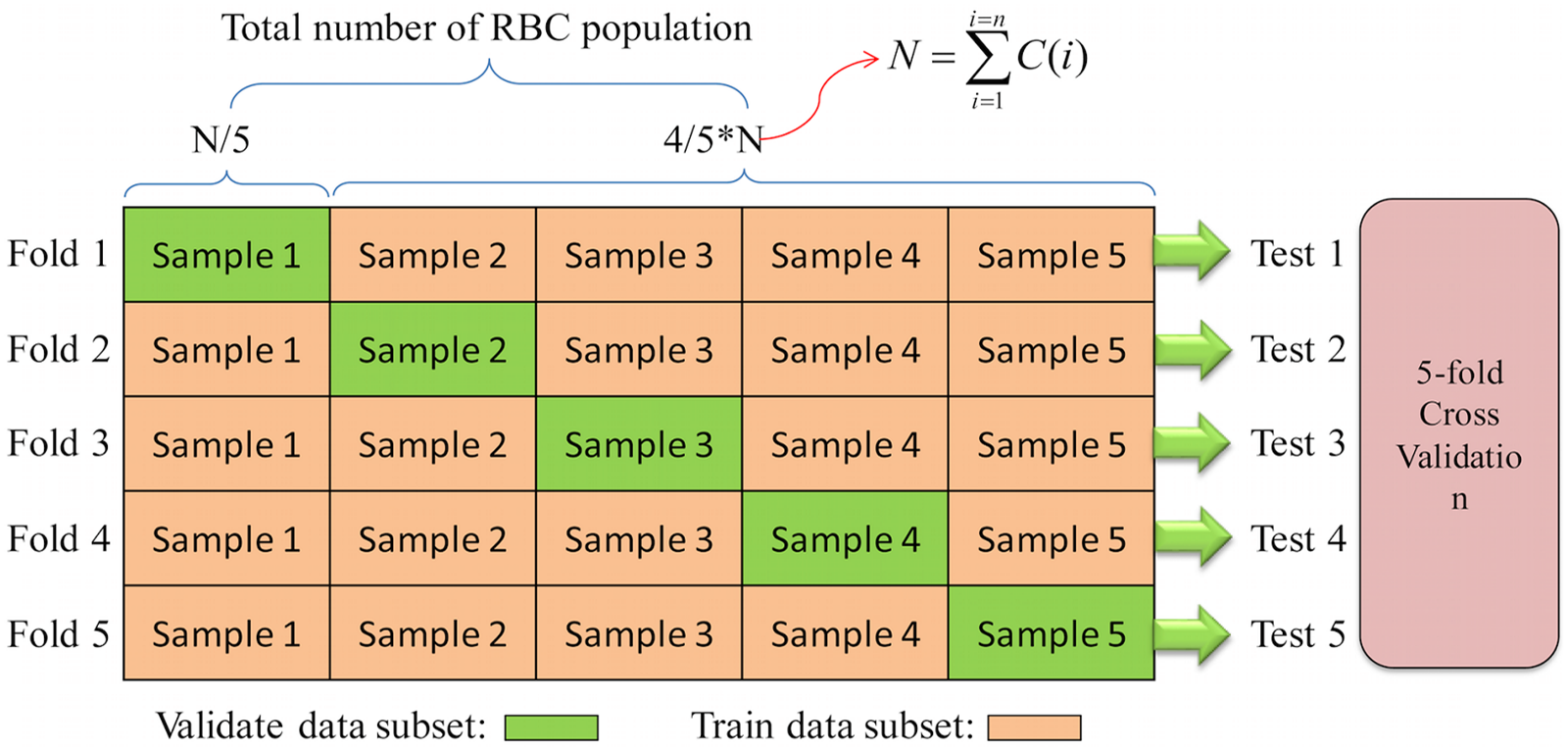
Evaluation procedure for 5-fold cross validation.

K-fold cross-validation is an effective way to measure the predictive performance for the deep CNNs model [29]. The K-fold cross-validation procedure is shown in Fig 11. First, the total RBC population was divided into *k* non-overlapped subsets with equal number of RBCs (here *k* was chosen to be 5). Then, for every fold or experiment, one of the 5 subsets was chosen as the validation set (green color data block) and the other *k* − 1 subsets were combined to form the training set (orange color data blocks). Finally, the average validation scores obtained from the five folds were calculated as the final prediction score. Every class of RBC images is divided into 5 equal subsets, the quantity of training data and validation data can in each class can be expressed as Eq (6).

$$\begin{array}{c} \left\{\begin{array}{c} \text{Sum}\left(V_{\text{ij}}\right)=C\left(i\right)/5,\\ \text{Sum}\left(T_{\text{ij}}\right)=1-C\left(i\right)/5, \end{array}i\in [1,n],j\in [1,5\right] \end{array}$$ (6)

Where, *C*(*i*) is the number of RBC in the *i*th class, *V*_*ij*_ describes the *i*-th class and *j*-th validation sub-dataset, and *T*_*ij*_ is the corresponding training dataset.*n* can take the values of 5 or 8 for our studies. Finally, 5 folds can be generated by collecting the same subset from different classes alternately. For example, the *j*-th fold can be represented by Eq (7).

$$\begin{array}{c} \text{fold}\left(j\right)=\{V_{1j},V_{2j},\ldots ,V_{nj}\}\cup \{T_{1j},T_{2j},\ldots ,T_{nj}\} \end{array}$$ (7)

The main advantage in using k-fold cross validation is that each image is limited to one use during the validation process. This can effectively avoid the inaccurate and unstable phenomenon while artificially forcing multiple common samples into both training and testing.

Hence, in order to evaluate the general performance of our RBC-dCNN model, we performed 5-fold cross validation for the new datasets Exp_II (7 patients), in which we created a supplement for the number of *echinocyte, granular, sickle* and *reticulocyte* categories. To evaluate the performance of our deep CNN model for the SCD RBC classification and determine the importance of different types of RBCs present in SCD blood, we perform the experiments according to the following principles:

1. Comparative study and analysis for two kinds of RBC labeling principles, denoted as “coarse labeling” and “refined labeling”, so as to validate the efficiency of the RBC-dCNN model applied in various kinds of RBC patterns in SCD.
2. Multiple metrics for the final evaluation of the performance of RBC-dCNN (Precision, Sensitivity, Specificity, F-score, Accuracy and ROC_AUC) are taken into account; the specifications for these measures are shown below.

$$\begin{array}{cc} & \text{Precision}=TP/(TP+FP) \end{array}$$ (8)

$$\begin{array}{cc} & \text{Sensitivity}=TP/(TP+FN) \end{array}$$ (9)

$$\begin{array}{cc} & \text{Specificity}=TN/(FP+TN) \end{array}$$ (10)

$$\begin{array}{cc} & F-\text{score}=2*TP/(2*TP+FP+FN) \end{array}$$ (11)

$$\begin{array}{cc} & \text{Accuracy}=(TP+TN)/(TP+FP+FN+TN) \end{array}$$ (12)

Here, *TP*, *TN*, *FP* and *FN* are, respectively, the true positive, true negative, false positive and false negative number of RBCs being classified for each class. The above five metrics can help us measure the dCNN’s performance from different perspective; e.g., the precision, or positive predictive value (*PPV*) can be viewed as a measure of a classifiers exactness. A low precision can also indicate a large number of False Positives. The sensitivity—also called recall or true positive rate(*TPR*)– measures the proportion of positives that are correctly identified; it can be viewed as a measure of a classifiers completeness. A low recall indicates many False Negatives; the specificity (*SPC*)—also known as true negative rate(*TNR*)– measures the proportion of negatives that are correctly identified. F1-score considers both precision and recall; it gets the best accuracy when it reaches 1, worst corresponds to 0. The ROC-AUC curve is a plot for TPR and NPR (Negative Positive Rate), which is explained in the experiments below.

In the following, the experimental results based on 5-fold cross validation for the two kinds of labeling datasets are presented respectively.

**Evaluation of coarse-labeled RBC dataset (5 categories):** In this experiment, all RBC patch images were coarsely labeled into 5 categories: 1) Dic+Ovl, 2) Ech, 3) El+Sk, 4) Grl, 5) Ret. In accordance with the cross validation scheme in Fig 11, the divided 5-fold cross validation datasets for 5 types of RBC and their corresponding evaluation results are given in Table 3.

**Table 3:** 5-fold cross validation for 5 types of SCD RBC classification.

Fold No.	Training set	Evaluation set	Training error	Evaluation error
*Fold*1	5664	1416	9.14%	10.54%
*Fold*2	5664	1416	9.07%	11.16%
*Fold*3	5664	1416	8.52%	10.08%
*Fold*4	5670	1410	9.84%	11.27%
*Fold*5	5658	1422	8.39%	10.55%
**Mean Accuracy**			91.01%±0.33%	89.28%±0.24%

**Fig 12:**
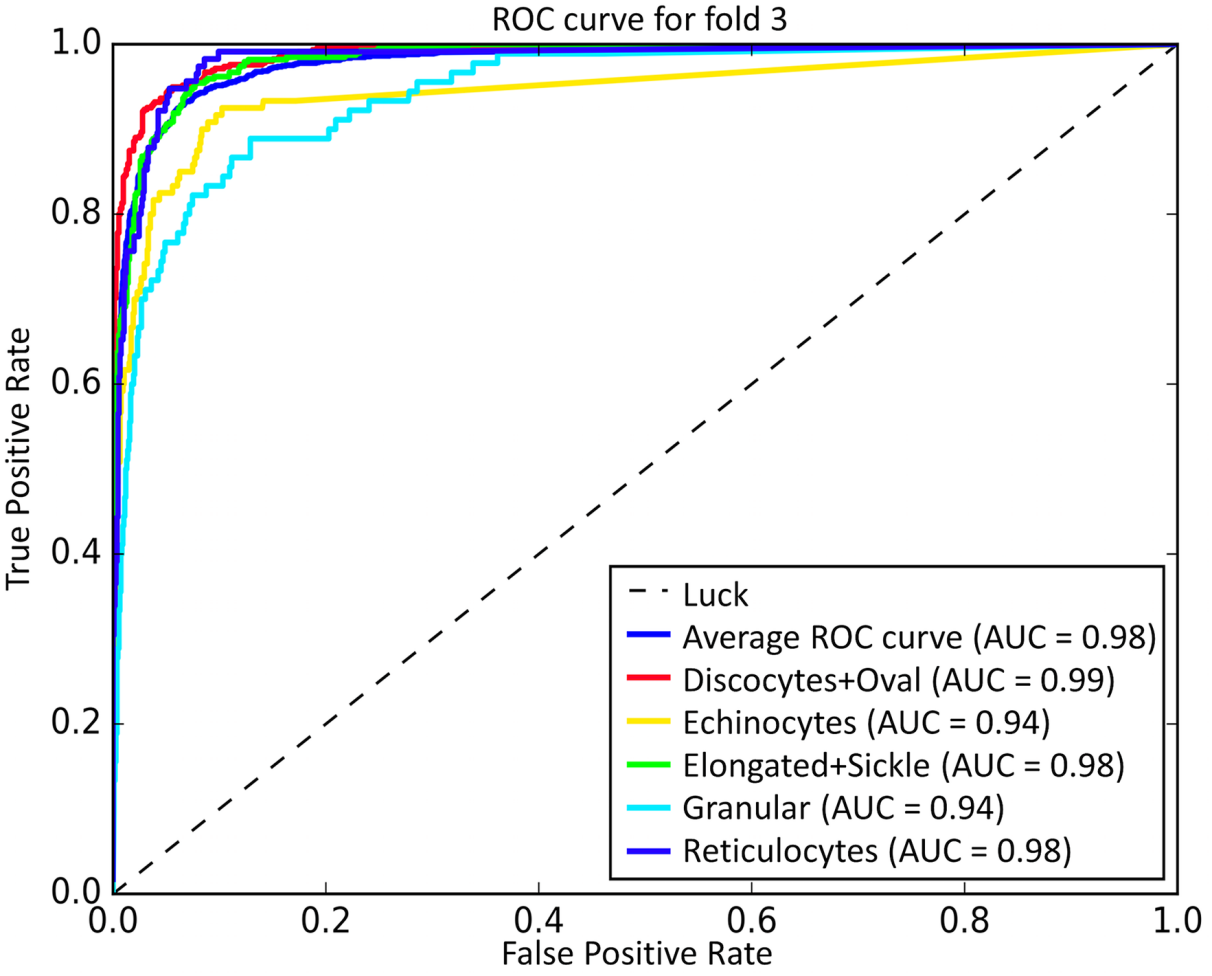
ROC-AUC result for coarse 5 types of RBC classification based on “Exp_II” dataset by 5-fold cross validation.

As seen from Table 3, the mean accuracy for training of 5 types of RBC classification under different folds is 91.01%, and the mean evaluation accuracy is 89.28%. Here, in order to better visualize the discriminative capability of the training deep CNNs model for RBC classification and investigate the sensitivity of the deep CNN model to various RBC categories, the Receiver Operating Characteristic (ROC) curve was used to plot the true positive rate (TPR) against false positive rate (FPR) for different classes of the 5-fold test. The top-left corner of a ROC plot is the “ideal point” while the diagonal dashed line indicates random chance or luck probability. Therefore, the closer the curve followed the left-top border of the ROC space, the more accurate the test can be considered. We also computed the AUC (Area Under the Curve) for each ROC curve to evaluate the prediction performance of our RBC-dCNN model. Fig 12 shows the corresponding ROC-AUC results for RBC classification with 5 target categories. In the ROC-AUC plot, the average ROC curve was calculated and shown in blue color, and the corresponding averaged AUC for each fold is at least 0.97. Regarding the prediction performance of the five RBC classes, Granular and Echinocytes received a relative low AUC value, and the other two classes (“Discocytes+Oval”and “Elongated+Sickle”) obtained a high AUC value.

**Fig 13:**
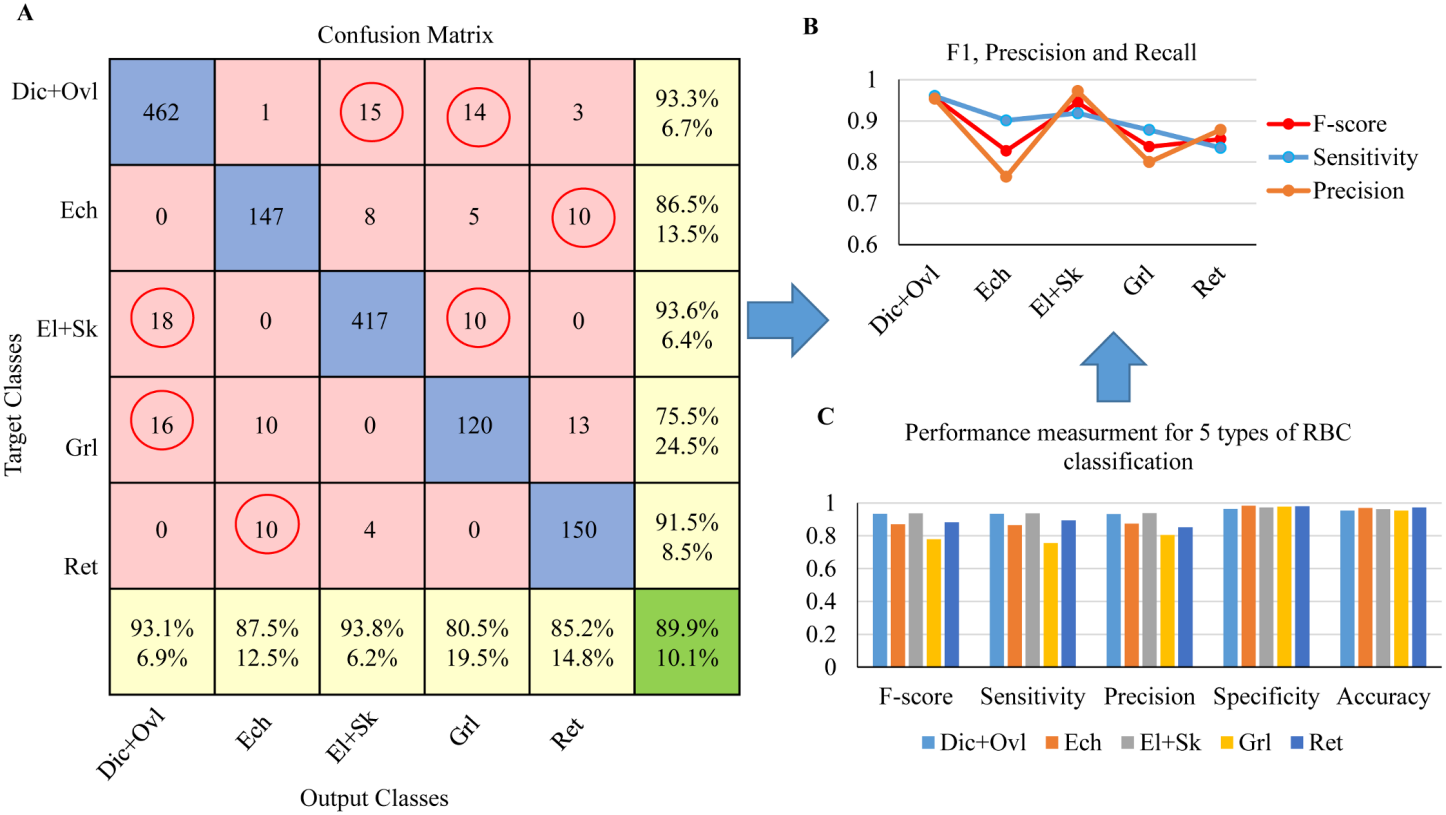
Performance analysis for SCD RBC classification based on “Exp_II” dataset (coarse labeling). (A) confusion matrix showing the detailed number of correctly classified RBC images and misclassified RBC images, (B) 5 statistic metrics for the 5 types of RBC category prediction results, (C) Specific F-score, precision and recall analysis for different RBC categories.

In addition, Fig 13A shows the corresponding confusion matrix, which can guide humans to observe the confusing classes in red circles; for instance, Ret and Ech are a pair of confusing classes, and the diagonal represents the correctly predicted number of each observation. The calculated sensitivity (right column) and precision (bottom) for each class in yellow color are consistent with the bars in the statistic Fig 13C. Except for these measures, three other measures are also computed for the performance analysis of the experiment, however, the difference in accuracy among the 5 type of RBCs is small because it refers to the true predictions (TP and TN) among the total validation dataset. However, high accuracy is not enough to demonstrate the goodness of the classifier, nor it can tell the sensitivity, precision, specificity and F-score. Therefore, it is necessary to explore these measures for a more in-depth analysis in the experiment. In Fig 13C, the Ret RBCs have a low recall (sensitivity), and the Ech get the lowest precision among the five classes. F-score can be applied to harmonize the above two evaluation metrics; the comparison results of F-score, precision and recall of 5 classes are shown in Fig 13B. Throughout all the evaluation measurements, we can obviously observe that the deep CNN model get a high accuracy and precision in predicting the different types of RBCs,in particular for “Dic+Ovl”, “El+Sk” and “Ret” types.

**Evaluation of refined-labeled RBC dataset (8 categories):** To evaluate the robustness of the deep CNN model in the application of more rich types of RBC classification, a refined labeling dataset “Exp_II” was generated, which included 8 types of RBC: *Dic, Ech, El, Grl, Ovl, Ret, Sk* and *Sto*. Similarly, 5-fold cross validation was carried out and the classification result is shown in Table 4. The mean evaluation accuracy for the 8 types of RBC classification was 87.50%.

**Table 4:** 5-fold cross validation for 8 types of SCD RBC classification.

Fold No.	Training set	Evaluation set	Training error	Evaluation error
*Fold*1	5772	1452	10.15%	12.41%
*Fold*2	5772	1452	11.02%	13.23%
*Fold*3	5772	1452	10.26%	12.99%
*Fold*4	5790	1434	10.13%	12.51%
*Fold*5	5790	1434	9.98%	11.38%
**Mean Accuracy**			89.69%±0.17%	87.50%±0.51%

**Fig 14:**
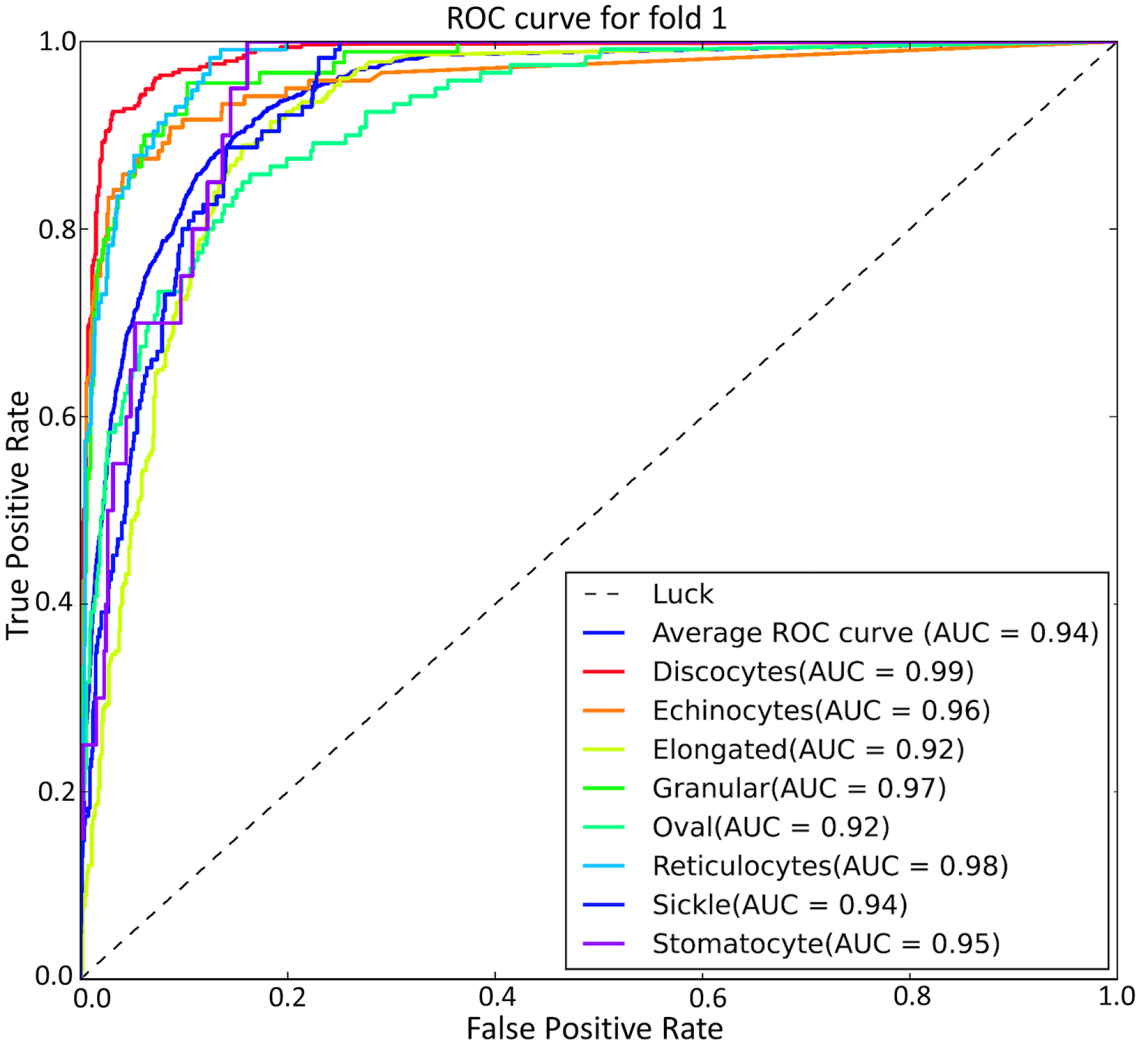
ROC-AUC result for refined 8 types of RBC classification based on “Exp_II” dataset by 5-fold cross validation.

**Fig 15:**
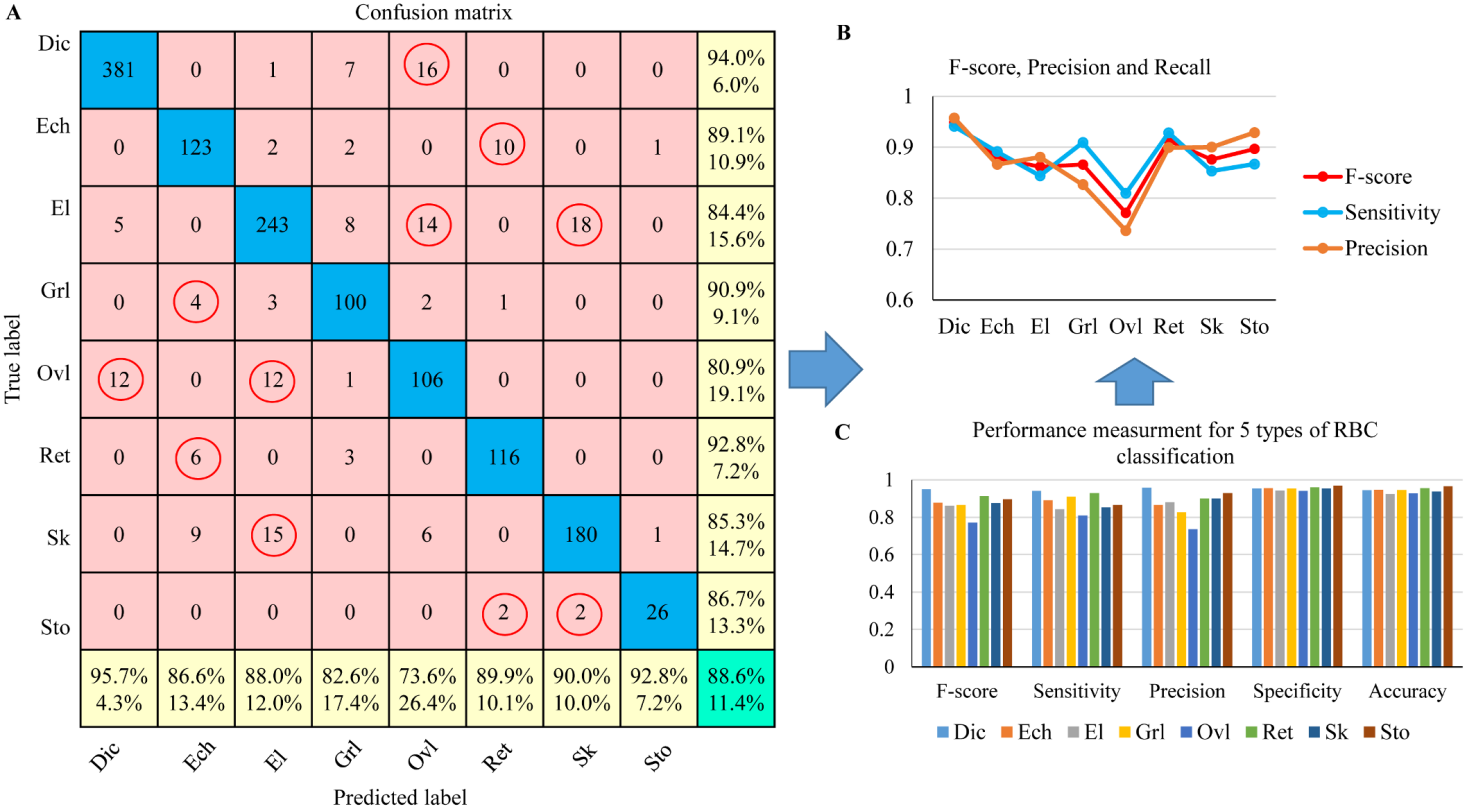
Classification performance analysis for SCD RBC classification based on “Exp_II” dataset (refined labeling). (A)confusion matrix showing the detailed number of correctly classified RBC images and misclassified RBC images. (B) 5 statistic metrics for the 8 RBC category prediction result. (C) Specific F-score, precision and recall analysis for different RBC categories.

The corresponding mean ROC-AUC result for the refined labeling test is shown in Fig 14. The average AUC value for 8 types of RBC is 0.94 as opposed to an average AUC value of 0.97 for the coarse labeling RBC classification. The RBC Categories (*El* and *Ovl*) got a relative low classification performance with an AUC value of 0.92. In addition, in Fig 15A, we see a more detailed confusion matrix for classification of 8 RBC categories. This shows the most confused classes (red circles) for each type of RBC; Fig 15b and 15c give a performance comparison among the 8 categories. *Dic* reached the best values for each metric and exhibited a sensitivity of 94.4% with high class-specific precision on testing sets of 1434 RBC images. *Ovl* type achieved the lowest precision and recall due to the misclassification with *Dic* and *El* types.

**Fig 16:**
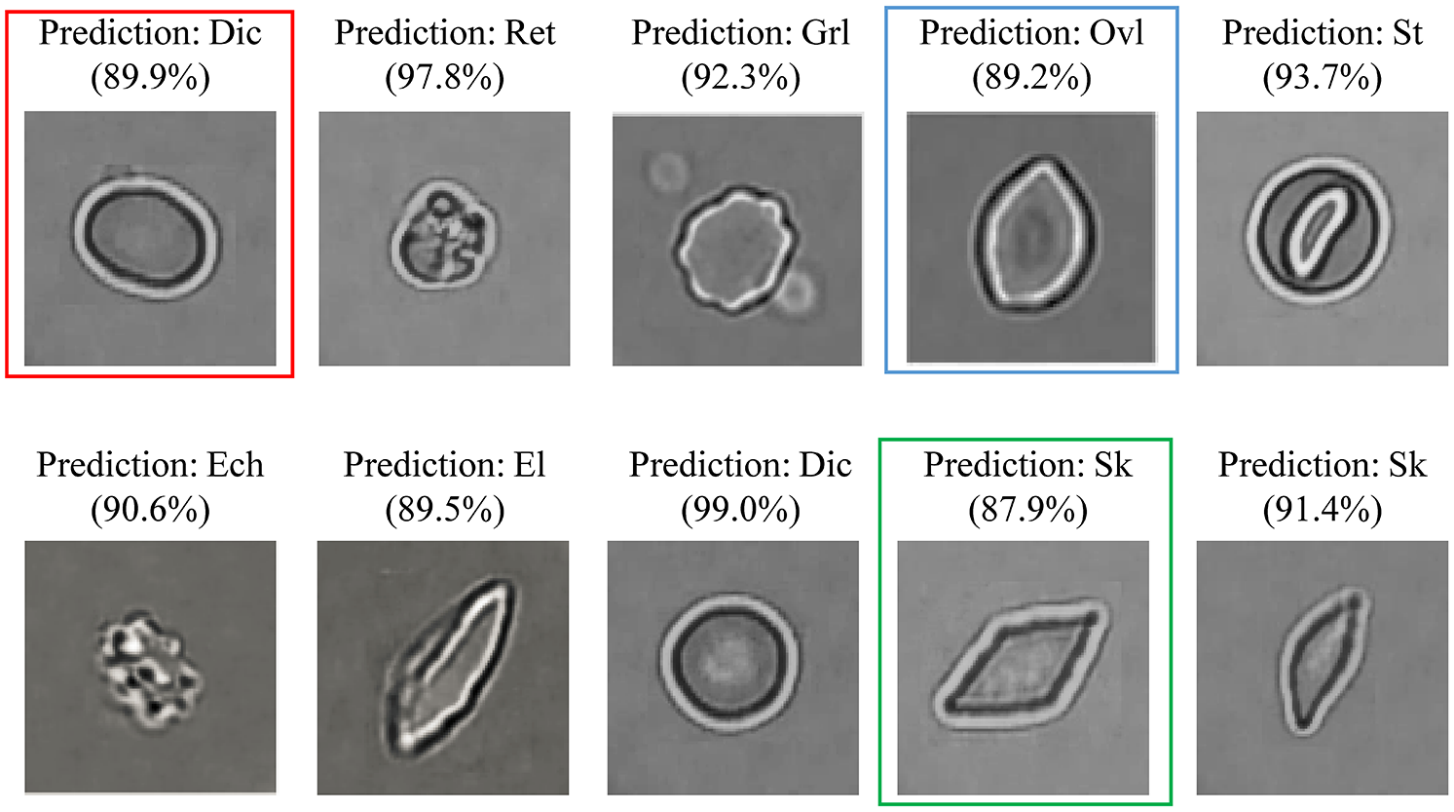
Prediction result example for 8 types of RBCs.

From the prediction result example in Fig 16 we can observe that some *Ovl* type RBCs (e.g. the RBC in red frame of Fig 16) are misclassified as *Dic* and the *El* type RBCs are prone to be classified to *Ovl* type and *Sk* type, e.g., the RBCs in blue and green frames in Fig 16.

**Fig 17:**
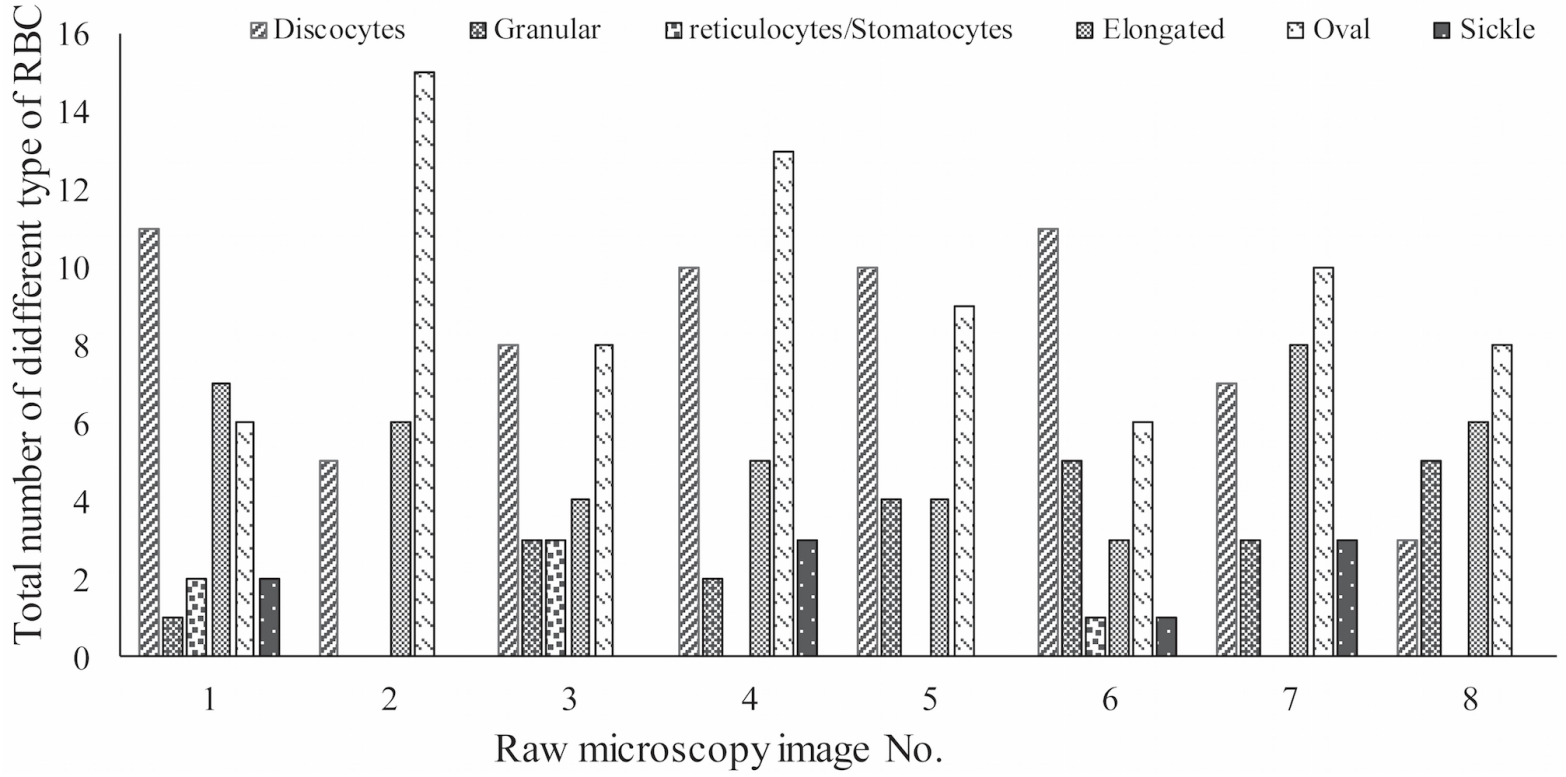
Statistical quantification for the number of various types of RBCs in density fraction 4 (severe SCD). Notice the significant heterogeneity of cell types even at the highest density fraction.

Based on the proposed deep RBC-CNN model, we perform an independent RBC classification test on 8 raw microscopy images in the highest density RBC fraction *i.e.* fraction 4 (typically associated with severe SCD). Statistical quantification results for the number of different types of RBC are shown in Fig 17. Notice the significant heterogeneity of cell types even at the highest density fraction.

### Classification of deoxygenated sickle RBCs

In addition to the above two experiments (EXP_I, EXP_II) on coarse-labeled and refine-labeled sickle RBC classification, in order to test the RBC-dCNN model for oxygenated and deoxygenated RBCs in SCD, we also performed a patient-specific experiment on the classification of a new experimental dataset that includes the previous coarse-labeled five catergories under normoxic conditions (Oxy) and a new catergory: “El+Sk under deoxygenation (DeOxy)”, see appendix for details on the experimental methodology. The specific experimental dataset (EXP_III) is shown in Table 5 (row 6) and it includes 81 El+Sk (DeOxy) RBCs, which after data augmentation correspond to an equivalent sample of 486 DeOxy RBCs.

**Table 5:** Description of our experimental dataset for the coarse-labeled five categories and a new deoxygenated category (row 6).

No.	RBC Types	Total number of RBCs	EXP_III (8 patients)
1	Dic+Ovl (Oxy)	456	2736
2	Ech (Oxy)	123	738
3	El+Sk (Oxy)	395	2370
4	Grl (Oxy)	91	546
5	Ret (Oxy)	115	690
6	El+Sk (DeOxy)	81	486
**Total number of RBCs**		1261	7566

**Fig 18:**
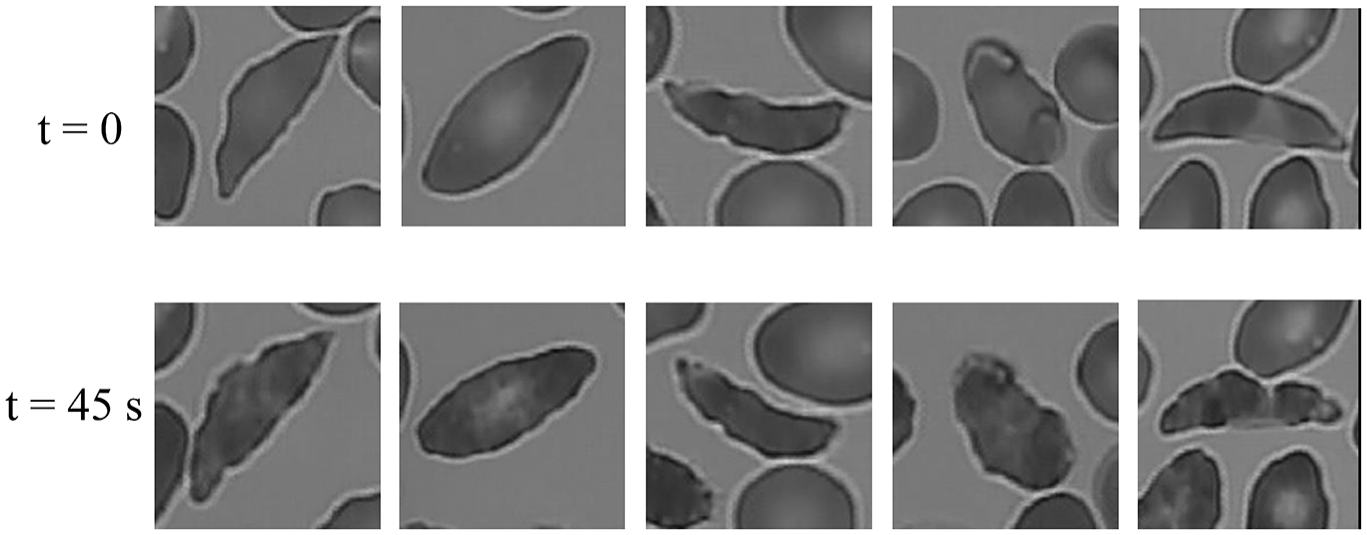
First row: (t = 0) Irreversibly sickled cells (ISCs) under normoxia i.e., Oxy state [*O*_2_]: 20%; Initiation of deoxygenation. Second row: (t = 45s) Deoxygenated ISCs i.e., DeOxy state [*O*_2_]: 2%; (see S4 Appendix methods on Oxy/DeOxy states).

In order to appreciate the differences in RBCs under Oxy and DeOxy conditions, we present in Fig 18 images of RBCs before and after deoxygenation. Even under Oxy, these particular RBCs have crenated shape because they are irreversibly sickled. However, upon deoxygenation we see that there is further polymerization of sickle hemoglobin (HbS) inside the RBCs, manifested by the roughening of the contours of RBCs as well as the alteration of the texture inside the RBCs. While the change in the overall shape of these deoxygenated RBCs is relatively small compared to their Oxy state, the differences are subtle and hence they present a new challenge for our dCNN.

**Fig 19:**
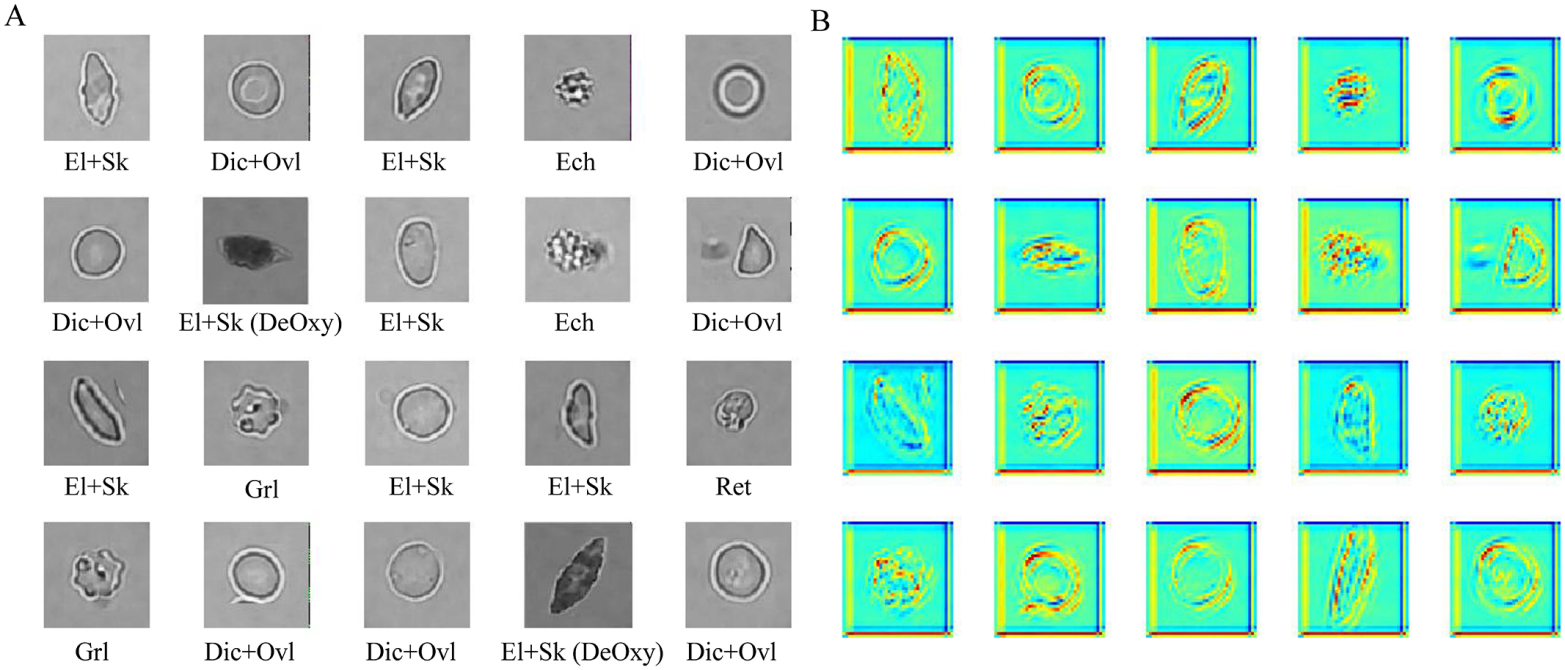
Snapshots of feature maps for layer 5 including the deoxygenated category. (**A**) original images in batch 1. (**B**) feature maps in 5th layer.

**Fig 20:**
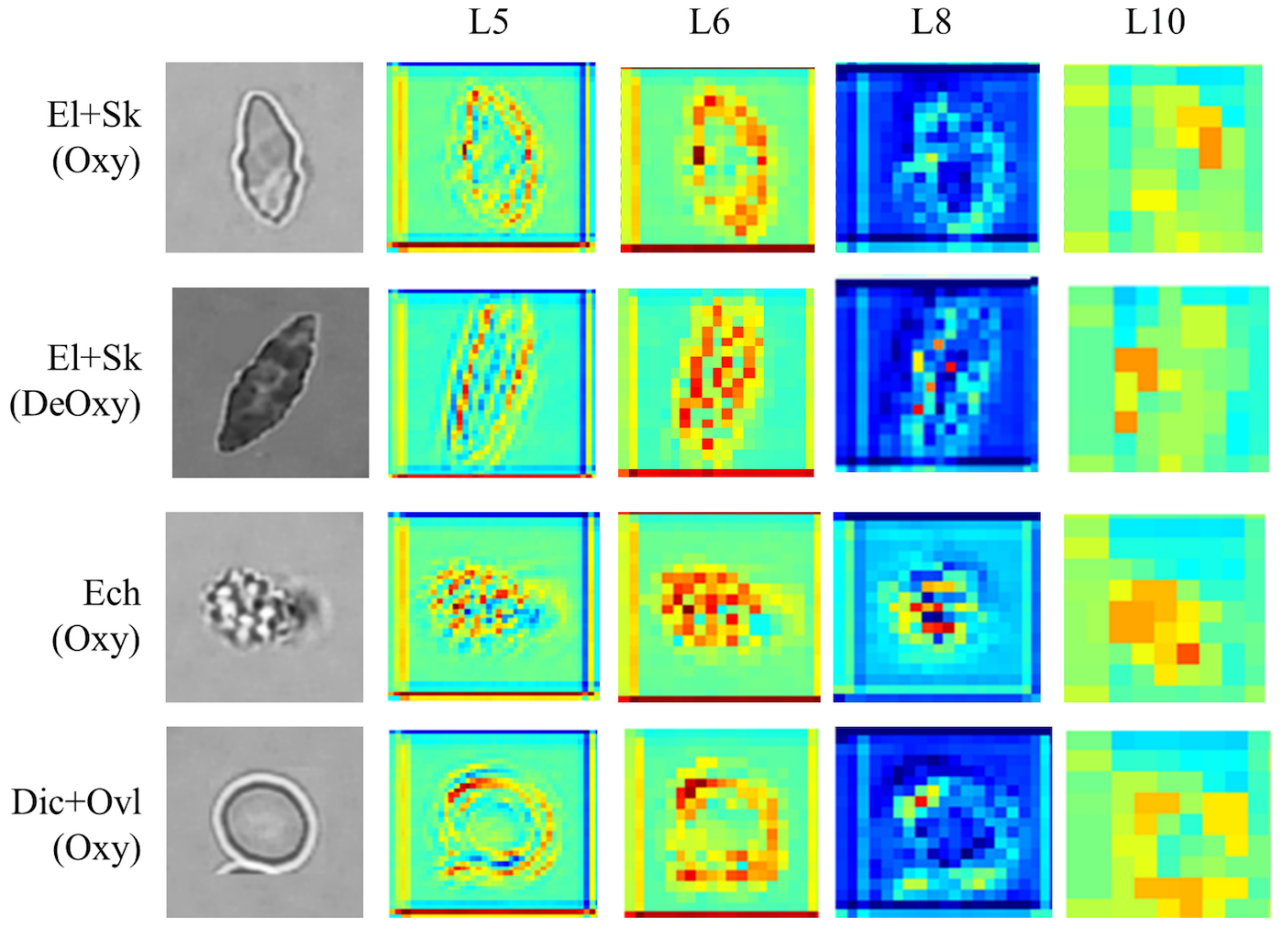
Learning hierarchical feature maps for layers 5, 6, 8, 10 for Dic+Ovl (Oxy), Ech (Oxy), El+Sk (Oxy), El+Sk (DeOxy).

Having this new mixed Oxy-DeOxy dataset (EXP_III) and the particular RBC inner pattern alteration characteristics, we carried out the dCNN model training and testing using the previous similar 5-fold cross validation schema, which involves four folds for training and one fold for testing. We have a total of 988 RBCs for training, which we arrange in 50 batches of 20 images each except the last one that has only 8 RBCs; see Fig 19A. So each batch contains 20 different RBCs, which may be in any of the six categories that the dCNN model should learn. The RBCs are randomly shuffled before input to dCNN. The hierarchical features can be extracted by dCNN layer-by-layer. For instance, the learned feature maps in the hidden 5th-layer for batch 1 is shown in Fig 19B. We observe that the convolutional operation can extract and highlight image features based on the raw image data field directly and hierarchically, such as detecting the image key points, edges, curves, etc. This is further illustrated in Fig 20, which presents a sequence of feature maps for different layers (layers 5, 6, 8 ad 10) corresponding to four different classes of RBCs in Oxy and DeOxy states. As we move to high layer numbers, we pick up more features from low level to high level, hence bridging the gap between high level representation and low level features. Within each layer different filters can be learned from the data to help extract different features. The images shown in Fig 20 correspond to arbitrary selection of filters for each layer. The original raw images are shown on the first column of Fig 20. Hence, the learned hierarchical convolutional features corresponding to variant learning filters play an important role in classifying RBCs in SCD, in particular for the classification of Oxy and irreversibly DeOxy sickle RBCs.

**Fig 21:**
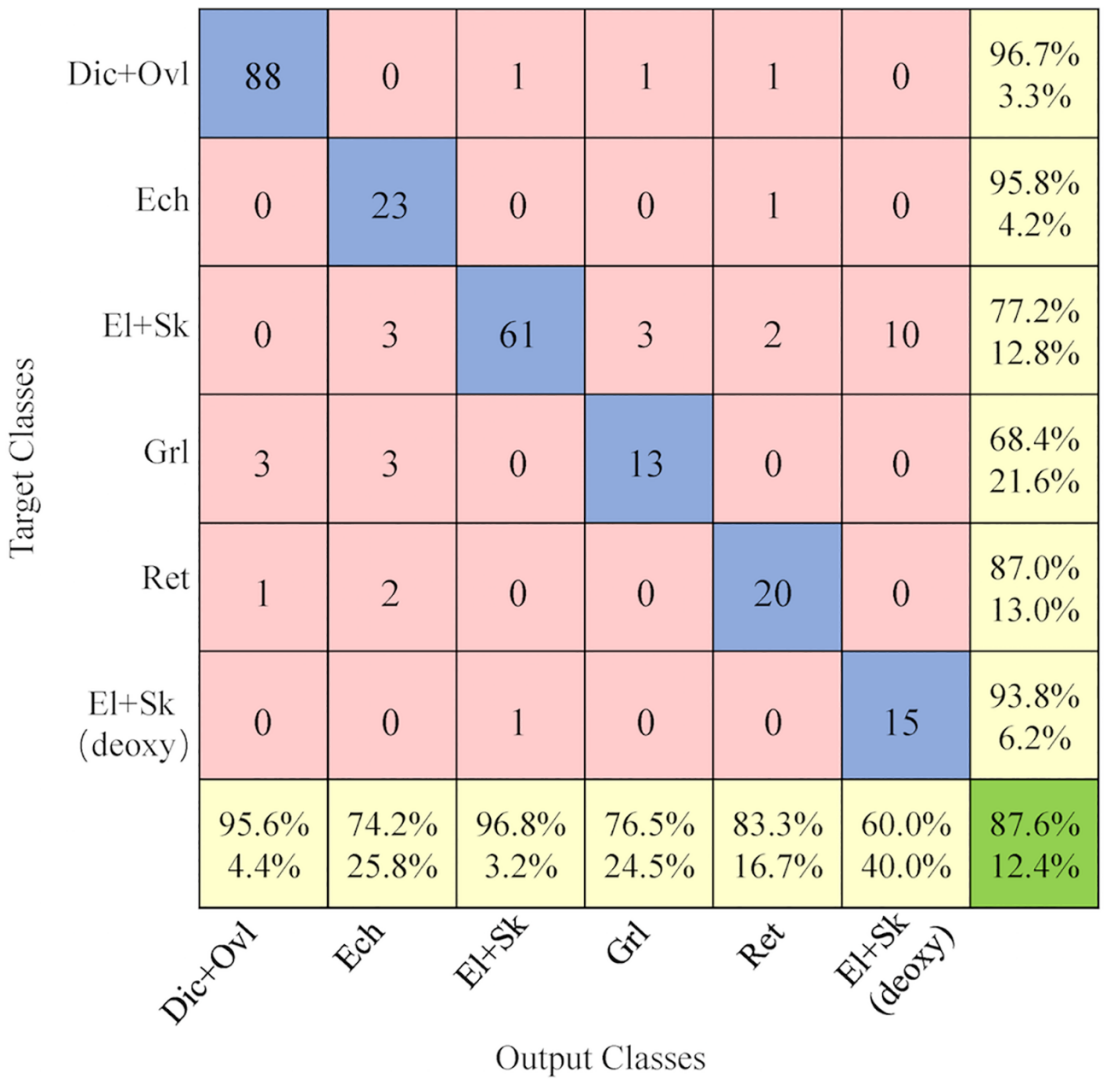
Confusion matrix for the combined oxygenated and deoxygenated categories described in Table 5.

The final prediction result for the classification of deoxygenated RBCs is shown in Fig 21 for the elongated and sickle (DeOxy) category. If there is an obvious intracellular pattern change, then the accuracy of our trained dCNN model can obtain a high recall (93.8%) but a relatively low precision (60.0%). The main reason for this phenomenon can be justified as follows:

1. The deoxygenated El+Sk RBCs with no obvious intracellular pattern alteration will be confused with the oxygenated El+Sk RBCs. Consequently, the accuracy of our deep CNN is relatively low. This is to be expected because those RBCs are deoxygenated, and as we already mentioned they do not change the shape significantly, so they look similar to El+Sk (Oxy) RBCs.
2. Initially we have hypothesized that there could be confusion between classes (Ech and El+Sk (DeOxy)) due to apparently similar patterns within the cells. However, surprisingly our trained dCNN (DeOxy) is able to distinguish the intracellular pattern with accuracy exceeding 67.5%.

Taken together, the above observations imply that both intracellular patterns and RBC contours play a significant role in classification (see Fig 18, second row).

### Shape factor quantification for classified RBCs

**Fig 22:**
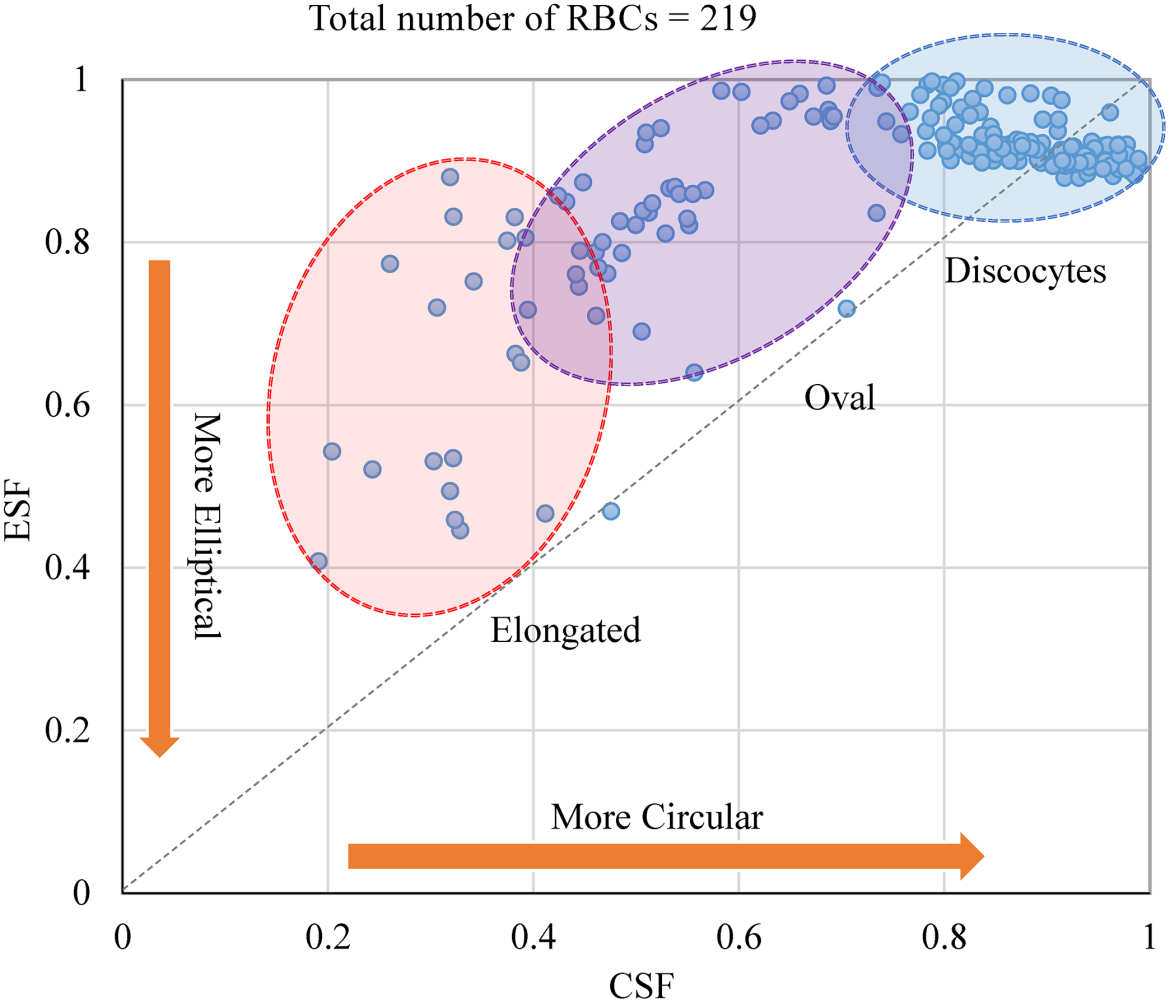
CSF and ESF shape factors estimation for Elongated, Oval and Discocytes RBC types.

Our proposed RBC classification methodology also relies on the extraction of individual RBCs shape factors that is complementary to RBC classification. Two of the most prevalent shape factors are the Circularity Shape Factor (CSF) and Ellipticity Shape Factor (ESF) (Also see S2 Appendix) [30–32]. We computed the CSF and ESF shape factors for the classified RBCs obtained with the RBC-dCNN methodology (see Fig 22). The graph is a statistical visual representation of the classified RBCs (*i.e.*, Elongated, Oval and Discocytes) within the ellipticity and circularity shape factor mapping. In addition to these two factors, we can implement in the workflow and compute any of the additional 12 shape factors mentioned in Table S-I to quantify SCD patient-specific RBC shape parameters. The results here are consistent with results described by Horiuchi et al. [30].

In summary, we have used patient-specific microscopy images to develop an automated, high-throughput, *ex-vivo* RBC classification method for the sickle cell disease based on pre-extraction of RBC region and deep CNNs. We employed a hierarchical RBC patch extraction method followed by a shape-invariant RBC patch normalization technique for the input of our deep nets, which can exclude unnecessary background patches and save time during both the training and the learning procedures. Moreover, our experiments for two kinds of labeling datasets (5 and 8 classes) based on different partition levels demonstrate the great capability and robustness of our RBC-dCNNs model on the classification of various RBC categories with characteristics of complex patterns and heterogeneous shapes without the need for hand-crafted feature pre-extraction. While most of the dCNN training was done based on oxygenated SCD RBCs, we also conducted the classification of deoxygenated RBCs, and demonstrate that our model can detect the deoxygenated RBCs with high accuracy capturing the subtle intracellular texture alterations. Furthermore, the explicit shape analysis at the end of the procedure can offer a robust morphological quantitative tool expanding the proposed framework to high-throughput, *ex-vivo* RBC classification.

Our program is written in Python language and C language, and it currently runs on CPUs, but it can also be updated to run on GPUs. It is mainly based on Python open-source libraries Theano, Numpy, SciPy and matplotlib, etc. The program takes only a few seconds on a standard desktop to test over a thousand RBCs using the trained deep neural network model.

In SCD, the shape of sickle RBCs is directly related to the polymerization process inside the RBC, which, in turn, depends on the de-oxygenation rate and hence the specific human organ where a sickle cell crisis may occur, consistent with clinical observations. The ability to perform high-throughput morphological classification utilizing deep CNNs of individual RBCs or other cell types, (*e.g.*, white blood cells) opens up complementary avenues in medical diagnostics for highly heterogeneous cell populations such as in hematological diseases and stored blood used for transfusion.

The framework presented here is powerful but many aspects can be further improved in future work. For example, new work should aim to: (1) develop an accurate segmentation method for the *overlapped* RBCs in the microscopy image; (2) increase the dataset scale on the number of rare categories, e.g. sickle, granular, stomatocytes, etc.; and (3) build a golden standard library containing diverse SCD RBC categories. Given the success of dCNN in classifying deoxygenated RBCs, having been trained mostly with oxygenated RBCs, we believe that with the proper training of dCNN, the overall methodology for classification we propose could be effective in other hematological disorders, e.g., diabetes mellitus, elliptocytosis, spherocytosis, as well as in classifying other cells, e.g., cancer cells, and even detecting the activation state of platelets.

## Supporting information

S1 Appendix RBC density fractionation and microscopy image data acquisition. (PDF)

S2 Appendix Automatic RBC shape factor quantification. It includes two parts: the specific computational formulas for the RBC shape factors and the corresponding pseudocode for RBC shape analysis.
(PDF)

S3 Appendix Review of deep convolutional neural network structure. (PDF)

S4 Appendix Deoxygenation methodology of sickle RBCs. (PDF)

## References

[1] Anglin C. Sickle Cell Disease. Journal of Consumer Health on the Internet. 2015;19(2):122–131. 10.1080/15398285.2015.1026706

[2] Fasano RM, Booth GS, Miles M, Du L, Koyama T, Meier ER, et al Red blood cell alloimmunization is influenced by recipient inflammatory state at time of transfusion in patients with sickle cell disease. British journal of haematology. 2015;168(2):291–300. 10.1111/bjh.13123 25256676

[3] Abubakar I, Tillmann T, Banerjee A. Global, regional, and national age-sex specific all-cause and cause-specific mortality for 240 causes of death, 1990-2013: a systematic analysis for the Global Burden of Disease Study 2013. Lancet. 2015;385(9963):117–171. 10.1016/S0140-6736(14)61682-2 25530442 PMC4340604

[4] Milton JN, Gordeuk VR, Taylor JG, Gladwin MT, Steinberg MH, Sebastiani P. Prediction of fetal hemoglobin in sickle cell anemia using an ensemble of genetic risk prediction models. Circulation: Cardiovascular Genetics. 2014; p. CIRCGENETICS–113. 10.1161/CIRCGENETICS.113.000387 PMC3994553 24585758

[5] Darrow MC, Zhang Y, Cinquin BP, Smith EA, Boudreau R, Rochat RH, et al Visualizing red blood cell sickling and the effects of inhibition of sphingosine kinase 1 using soft X-ray tomography. J Cell Sci. 2016;129(18):3511–3517. 10.1242/jcs.189225 27505892 PMC5047677

[6] van Beers EJ, Samsel L, Mendelsohn L, Saiyed R, Fertrin KY, Brantner CA, et al Imaging flow cytometry for automated detection of hypoxia-induced erythrocyte shape change in sickle cell disease. American journal of hematology. 2014;89(6):598–603. 10.1002/ajh.23699 24585634 PMC4180063

[7] He Y, Gong H, Xiong B, Xu X, Li A, Jiang T, et al iCut: an integrative cut algorithm enables accurate segmentation of touching cells. Scientific reports. 2015;5. 10.1038/srep12089 PMC4501004 26168908

[8] Liu Z, Liu J, Xiao X, Yuan H, Li X, Chang J, et al Segmentation of White Blood Cells through Nucleus Mark Watershed Operations and Mean Shift Clustering. sensors. 2015;15(9):22561–22586. 10.3390/s150922561 26370995 PMC4610533

[9] Arteta C, Lempitsky V, Noble JA, Zisserman A. Detecting overlapping instances in microscopy images using extremal region trees. Medical image analysis. 2016;27:3–16. 10.1016/j.media.2015.03.002 25980675

[10] Arteta C, Lempitsky V, Noble J, Zisserman A. Learning to detect cells using non-overlapping extremal regions. Medical image computing and computer-assisted intervention–MICCAI 2012. 2012; p. 348–356. 10.1007/978-3-642-33415-3_43 23285570

[11] Carpenter AE, Jones TR, Lamprecht MR, Clarke C, Kang IH, Friman O, et al CellProfiler: image analysis software for identifying and quantifying cell phenotypes. Genome biology. 2006;7(10):R100 10.1186/gb-2006-7-10-r100 17076895 PMC1794559

[12] Sacan A, Ferhatosmanoglu H, Coskun H. CellTrack: an open-source software for cell tracking and motility analysis. Bioinformatics. 2008;24(14):1647–1649. 10.1093/bioinformatics/btn247 18511469

[13] Schindelin J, Arganda-Carreras I, Frise E, Kaynig V, Longair M, Pietzsch T, et al Fiji: an open-source platform for biological-image analysis. Nature methods. 2012;9(7):676–682. 10.1038/nmeth.2019 22743772 PMC3855844

[14] Hodneland E, Kögel T, Frei DM, Gerdes HH, Lundervold A. CellSegm-a MATLAB toolbox for high-throughput 3D cell segmentation. Source code for biology and medicine. 2013;8(1):16 10.1186/1751-0473-8-16 23938087 PMC3850890

[15] Shekhar K, Brodin P, Davis MM, Chakraborty AK. Automatic classification of cellular expression by nonlinear stochastic embedding (ACCENSE). Proceedings of the National Academy of Sciences. 2014;111(1):202–207. 10.1073/pnas.1321405111 PMC3890841 24344260

[16] Liu L, Wang L. HEp-2 cell image classification with multiple linear descriptors. Pattern Recognition. 2014;47(7):2400–2408. 10.1016/j.patcog.2013.09.022

[17] Liu A, Gao Z, Tong H, Su Y, Yang Z. Sparse coding induced transfer learning for hep-2 cell classification. Bio-medical materials and engineering. 2014;24(1):237–243. 24211903 10.3233/BME-130804

[18] Donato C, Vincenzo T, Marco C, Francesco F, Maria VS, Giuseppe R. HEp-2 cell classification with heterogeneous classes-processes based on k-nearest neighbours. In: Pattern Recognition Techniques for Indirect Immunofluorescence Images (I3A), 2014 1st Workshop on. IEEE; 2014. p. 10–15.

[19] Gao Z, Zhang J, Zhou L, Wang L. Hep-2 cell image classification with convolutional neural networks. In: Pattern Recognition Techniques for Indirect Immunofluorescence Images (I3A), 2014 1st Workshop on. IEEE; 2014. p. 24–28.

[20] Li H, Zhang J, Zheng WS. Deep CNNs for HEp-2 Cells Classification: A Cross-specimen Analysis. arXiv preprint arXiv:160405816. 2016;.

[21] Hosseini P, Abidi SZ, Du E, Papageorgiou DP, Choi Y, Park Y, et al Cellular normoxic biophysical markers of hydroxyurea treatment in sickle cell disease. Proceedings of the National Academy of Sciences. 2016; p. 201610435. 10.1073/pnas.1610435113 PMC5003247 27512047

[22] Han XH, Lei J, Chen YW. HEp-2 Cell Classification Using K-Support Spatial Pooling in Deep CNNs. In: International Workshop on Large-Scale Annotation of Biomedical Data and Expert Label Synthesis. Springer; 2016. p. 3–11.

[23] Grady L. Random walks for image segmentation. IEEE transactions on pattern analysis and machine intelligence. 2006;28(11):1768–1783. 10.1109/TPAMI.2006.233 17063682

[24] Dokmanic I, Parhizkar R, Ranieri J, Vetterli M. Euclidean distance matrices: essential theory, algorithms, and applications. IEEE Signal Processing Magazine. 2015;32(6):12–30. 10.1109/MSP.2015.2398954

[25] Ozpolat HT, Chang T, Chen J, Wu X, Norby C, Konkle BA, et al Evaluation of Cell Types and Morphologies in Sickle Cell Disease with an Imaging Flow Cytometer. Blood. 2015;126(23):972–972.26022238

[26] Hiruma H, Noguchi CT, Uyesaka N, Hasegawa S, Blanchette-Mackie EJ, Schechter AN, et al Sickle cell rheology is determined by polymer fraction–not cell morphology. American journal of hematology. 1995;48(1):19–28. 10.1002/ajh.2830480105 7832188

[27] HW GL, Wortis M, Mukhopadhyay R. Stomatocyte–discocyte–echinocyte sequence of the human red blood cell: Evidence for the bilayer–couple hypothesis from membrane mechanics. Proceedings of the National Academy of Sciences. 2002;99(26):16766–16769. 10.1073/pnas.202617299 PMC139218 12471152

[28] Srivastava N, Hinton GE, Krizhevsky A, Sutskever I, Salakhutdinov R. Dropout: a simple way to prevent neural networks from overfitting. Journal of Machine Learning Research. 2014;15(1):1929–1958.

[29] Wong TT. Performance evaluation of classification algorithms by k-fold and leave-one-out cross validation. Pattern Recognition. 2015;48(9):2839–2846. 10.1016/j.patcog.2015.03.009

[30] Hijiya N, Horiuchi K, Asakura T. Morphology of sickle cells produced in solutions of varying osmolarities. The Journal of laboratory and clinical medicine. 1991;117(1):60–66. 1987310

[31] Asakura T, Mattiello JA, Obata K, Asakura K, Reilly MP, Tomassini N, et al Partially oxygenated sickled cells: sickle-shaped red cells found in circulating blood of patients with sickle cell disease. Proceedings of the National Academy of Sciences. 1994;91(26):12589–12593. 10.1073/pnas.91.26.12589 PMC45484 7809083

[32] Asakura T, Asakura K, Obata K, Mattiello J, Ballas SK. Blood samples collected under venous oxygen pressure from patients with sickle cell disease contain a significant number of a new type of reversibly sickled cells: Constancy of the percentage of sickled cells in individual patients during steady state. American journal of hematology. 2005;80(4):249–256. 10.1002/ajh.20468 16315254

