# Supplementary material for: A deep convolutional neural network for classification of red blood cells in sickle cell anemia
Source: PLoS Comput Biol. 2017 Oct 19;13(10):e1005746. doi: 10.1371/journal.pcbi.1005746 (PMC5654260; doi:10.1371/journal.pcbi.1005746)
Supplement: S1 Appendix — (PDF) [file pcbi.1005746.s001.pdf]

Appendix S1. RBC density fractionation and microscopy image data acquisition

Samples were obtained from seven SCD patients from Massachusetts General Hospital (MGH) and University of Pittsburgh Medical Center (UPMC) in accordance with institutional review board. Blood samples were stored at 4 °C and utilized within 3 days following blood draw.

Density-fractionated labeling of RBCs for CNN training: Density fractionation was performed to aid the accuracy of the manual RBC labeling for the deep CNN training. This was done by subdividing blood samples from SCD patients into four density fractions as it is illustrated in Fig S1. RBCs with shapes that deviate the most from the normal biconcave shape (so-called discocyte shape in 2D) tend to occur in the denser populations; they are generally absent in healthy human blood. It has been suggested that such cells have been irreversibly damaged either through aging or repeated cycles of oxygenation and deoxygenation during blood circulation. RBC damage is reflected in the deviations of the discoid shape e.g. oval, elongated, sickle RBC shapes. The density-fractionation process was described in detail in [1]. Briefly, packed RBCs after washing and fractionation were re-suspended at 1% hematocrit in a phosphate buffered saline (PBS) solution with 1% bovine serum albumin (BSA) and imaged within an hour. RBCs were imaged on a Zeiss inverted Axiovert 200 microscope under 63x oil objective lens using an Industrial camera (Sony Exmor CMOS color sensor) under 1080p resolution for image acquisition.

References

1. Hosseini P, Abidi SZ, Du E, Papageorgiou DP, Choi Y, Park Y, et al. Cellular normoxic biophysical markers of hydroxyurea treatment in sickle cell disease. Proceedings of the National Academy of Sciences. 2016; p. 201610435.

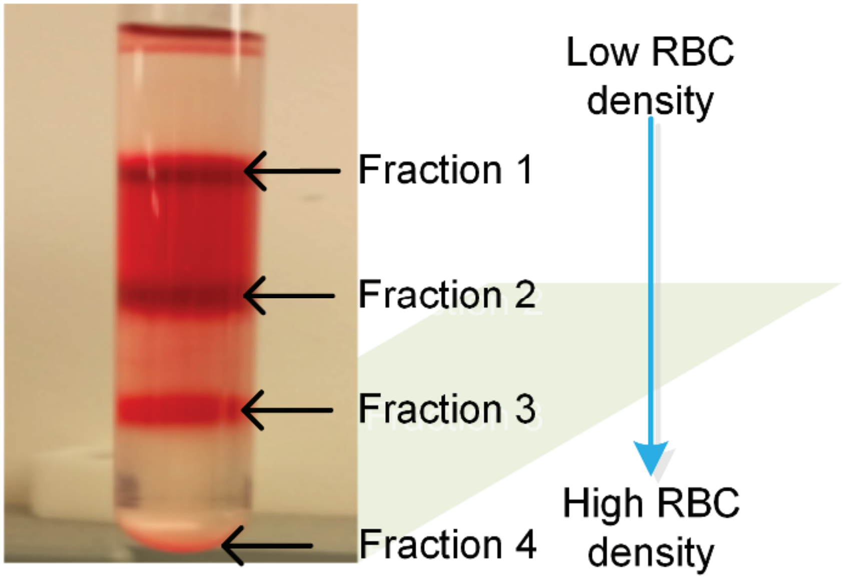

(a)

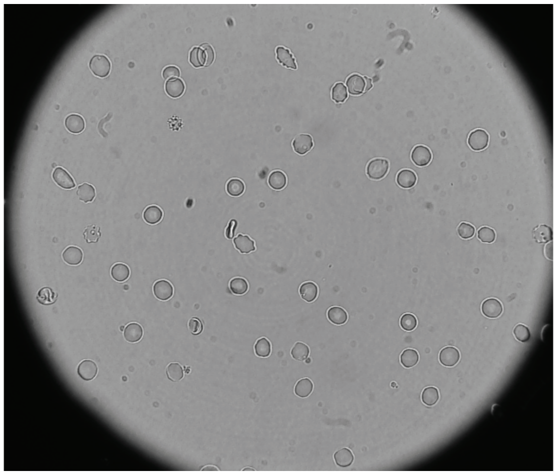

(b)

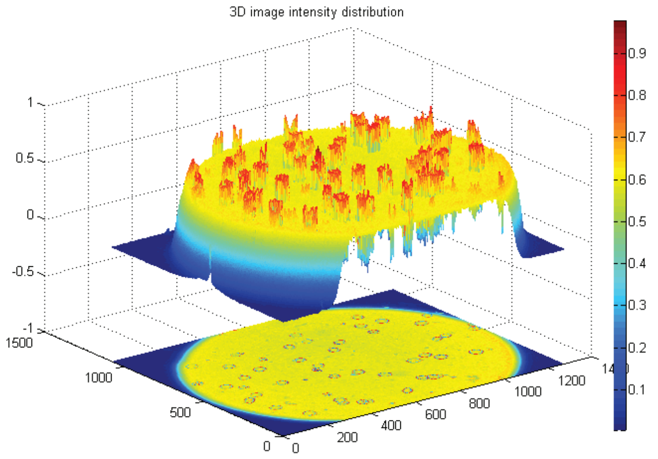

(c)

Figure S1: **Experimental characterization of sickle RBCs.** (a) density fractionation result from SCD blood. Increasing RBC density is indicated with the blue arrow. (b), (c) Representative raw microscopy image obtained from the microscope camera and corresponding intensity distribution.
