# Supplementary material for: A deep convolutional neural network for classification of red blood cells in sickle cell anemia
Source: PLoS Comput Biol. 2017 Oct 19;13(10):e1005746. doi: 10.1371/journal.pcbi.1005746 (PMC5654260; doi:10.1371/journal.pcbi.1005746)
Supplement: S2 Appendix — It includes two parts: the specific computational formulas for the RBC shape factors and the corresponding pseudocode for RBC shape analysis. (PDF) [file pcbi.1005746.s002.pdf]

Appendix S2. Automatic RBC shape factor quantification

In this work we conducted RBC shape quantification after the RBCs were classified by the deep CNN into their respective categories. We divided the shape factors into three main types: Elliptical, Regional and Derived shape factors. Table S1 describes the complete shape factors and their formulas, and Table S2 gives a pseudo-code for the RBC shape analysis method. It consists of four main steps:

- Load the RBC image to be analyzed (line 3);
- RBC outline detection for the RBC images (line 5);
- Calculate three kinds of shape factors (line 6 to 16);
- Save the calculated shape factor result into excel file (line 18 to 20).

In particular, *segOutline* is a function that implements the RBC segmentation by using the method described in Section B of the main text. The outputsRBC outline image (Bw) and binary label image (L) correspond to the extracted RBC outline and label image; calRegionprops is a function for calculating the regional properties ($Area\_r$, $Perm\_r$, $Area\_c$ and $Perm\_c$) of binary RBC mask image L; *calFeretDiameter* is applied to calculate the maxFD and minFD; *fitEllipse* is a function conducting ellipse fitting for the RBC contour, so as to generate the ellipse shape factors (Rb, Ra, θ and $Centroid$). The other 7 shape factors in Table S1 are derived from the regional shape indices and elliptical fitting shape indices. Finally, all the calculated 14 shape factors are appended into a structure type variable F through the *appendFactors* function for each RBC, and *save2Excelfile* is used to save all RBCs shape factor values into a single excel file.

Table S1: **Comparisons of loss and train errors based on different iterations.**

Types of Shape factors	Shape factors	Formula
**Elliptical shape factors**	Short axis(Rb)<br>long axis(Ra)<br>rotation angle (θ)<br>centroid	Based on the fitted ellipse result
**Regional shape factors**	segmented area and perimeter of RBC ($Area\_r$, $Perm\_r$)<br>convex area and perimeter ($Area\_c$, $Perm\_c$)<br>maximum Feret diameter ($maxFD$)<br>minimum Feret diameter ($minFD$)	Based on the fitted ellipse and convex hull polygon results
**Derived shape factors**	Elliptical shape factor(ESF)<br>Circular shape factor(CSF)<br>SF1<br>SF2<br>Elongation<br>Convexity<br>Compactness	$CSF = 4\pi * Area\_r/Perm\_r^2$<br>$ESF = Rb/Ra$<br>$SF1 = Rb/max\_FD$<br>$SF2 = min\_FD/max\_FD$<br>$Elongation = max\_FD/min\_FD$<br>$conv = \sqrt{Area\_r/Area\_c}$<br>$compt = 4\pi * Area\_r/Perm\_c^2$

Table S2. Illustration of the pseudocode for RBC shape analysis.

Algorithm 1: RBC_ShapeAnalysis

```
input  : RBC patch image location P
output: Shape factors F
begin
    rowstart ← 1
    [patch_img, cell_counter, patch_name] ← load_data(P)
    for i ∈ cell_counter do
        [Bw, L] ← segOutline(patch_img{i})
        [Area_r, Perm_r, Area_c, Perm_c] ← calRegionprops(L)
        [maxFD, minFD] ← calFeretDiameter(L)
        point_list ← find(Bw == 0)
        [centroid, Ra, Rb, θ] ← fitEllipse(point_list)
        ESF ← Rb/Ra
        CSF ← (4πArea_r)/Perm_r^2
        SF1 ← Rb/maxFD
        SF2 ← minFD/maxFD
        Elongation ← maxFD/minFD
        Conv ← sqrt(Area_r/Area_c)
        Compt ← (4πArea_r)/Perm_c^2
        F[i].factors ← appendFactors(centroid, Ra, Rb, θ, ESF, CSF,
        Perm_r, maxFD, minFD, SF1, SF2, Elongation, Conv, Compt);
    for i ∈ cell_counter do
        save2Excelfile(F[i].factors, rowstart, patch_name[i])
        rowstart ← rowstart + 1
```
