# Supplementary material for: A deep convolutional neural network for classification of red blood cells in sickle cell anemia
Source: PLoS Comput Biol. 2017 Oct 19;13(10):e1005746. doi: 10.1371/journal.pcbi.1005746 (PMC5654260; doi:10.1371/journal.pcbi.1005746)
Supplement: S3 Appendix — (PDF) [file pcbi.1005746.s003.pdf]

Appendix S3. Review of deep convolutional neural network structure

The deep CNN method can extract image features hierachically (features refer to details of images, e.g. pixels, edge, texton, motif, part and the whole object) through multiple layers of processing units (called hidden layers) between the input and output. It mainly contains three types of layers in the deep CNN:

1) Convolutional layer: it is the core layer in the architecture of CNN. A set of learnable filters (kernels or feature detectors) are convolved with the overlapped subsets of the input across the width and height. The output feature maps (or activation maps) corresponding to the convolutional layer are generated by adding a bias and applying a non-linear activation function (e.g., sigmoid, tanh, rectified linear unit (ReLU), etc.). The detailed expression for the i-th feature map at layer k, denoted as Z_i^k , can be expressed as:

$$\mathrm{Z_i^k} = J(\sum_{j=1}^{n_{k-1}} w_{ji}^k * Z_j^k - 1 + b_i^k), \tag{1}$$

where i and j are, respectively, the feature map index at layer k and layer $k-1$, J is the non-linear activation function (ReLU activation function– $max(0, x)$ is applied in this paper), w_{ji}^k denotes the kernel shared in the i-th feature map at layer k , and b_i^k is the corresponding bias of the i-th feature map at layer k. Notice that each feature map shares the same parameterization (weights and bias) for different receptive field, as shown in Fig S2, thus, it can effectively detect features regardless of their position and reduce the number of parameters to be learned in the training stage. The total number of learnable parameters is $(h*h*r+1)*n_k$, and the output size of feature map is denoted by $(W - h)/S + 1$, where S is the stride (in Fig S2, stride $S = 1$). The depth for the output feature map is decided by the number of total filters.

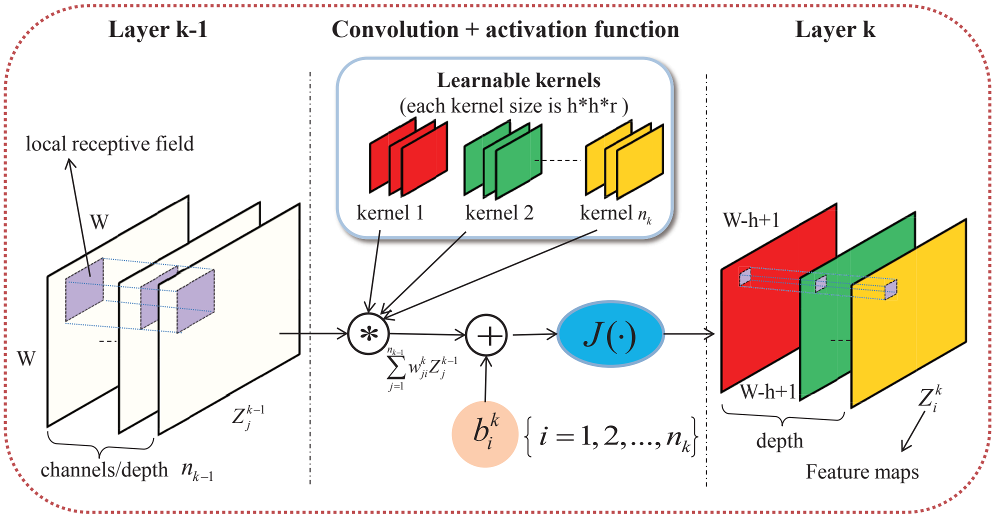

Figure S2: **Convolutional layer diagram showing two adjacent layers (left and right) separated by the sketch of the specific convolution process.**

2) Pooling layer: It is also known as sub-sampling or down-sampling layer, which is generally inserted periodically between successive convolutional layers. This technique can help reduce the spatial dimension of output feature maps, and thereby gradually decrease the amount of parameters to learn in different layers. Furthermore, it will result in traslation invariant features and enable training the CNN more efficiently. So far, there are 3 commonly used pooling methods [1]: max-pooling, mean pooling, and stochastic pooling (e.g., the factional max-pooling [2]), which allows to downsample the feature map with a non-integer multiplicative factor. However, this method needs more computational effort. In our study, we choose the most efficient max-pooling method to improve the convolved feature map subsampling speed. The corresponding diagram is given in Fig S3.

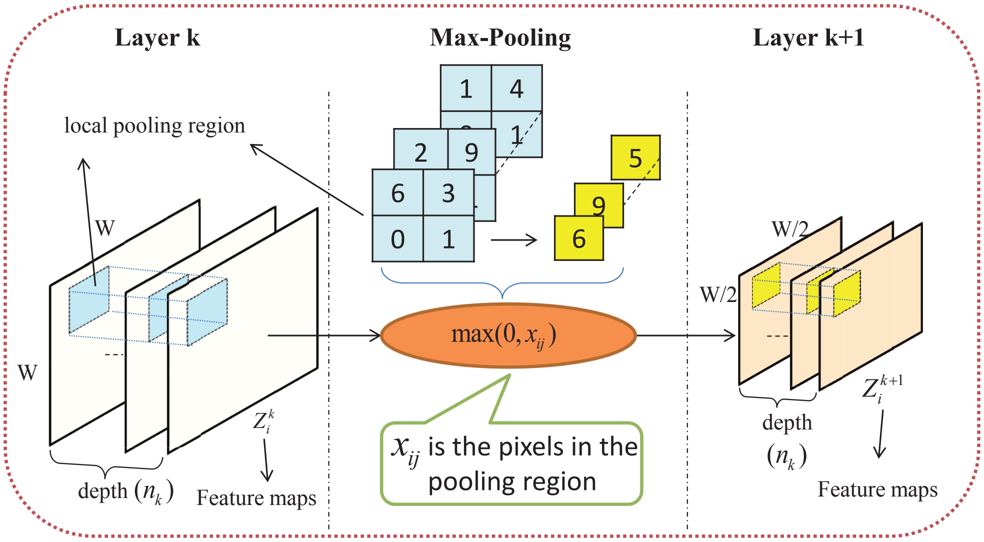

Figure S3: **Max-Pooling layer diagram showing two adjacent layers (left and right) separated by the sketch of the specific max-pooling process.**

Max-pooling operations are adopted to capture the strongest activation of the filter template with the input for each region. The non-overlapped subregion in blue color at the previous layer is eventually down-sampled to one single value (in yellow color) via the maximum response operation. The depth of output feature maps in the pooling layers still remains the same as in the previous layer.

3) Fully connected layer: Usually, after several convolutional layers and pooling layers all neurons in the previous layer are connected to every single neuron in the next layer. Consequently, the fully connected layer generates a one-dimensional feature vector.

References

1. Zeiler MD, Fergus R. Stochastic pooling for regularization of deep convolutional neural networks. arXiv preprint arXiv:13013557. 2013;.
2. Graham B. Fractional max-pooling. arXiv preprint arXiv:14126071. 2014;.
