# Supplementary material for: A deep convolutional neural network for classification of red blood cells in sickle cell anemia
Source: PLoS Comput Biol. 2017 Oct 19;13(10):e1005746. doi: 10.1371/journal.pcbi.1005746 (PMC5654260; doi:10.1371/journal.pcbi.1005746)
Supplement: S4 Appendix — (PDF) [file pcbi.1005746.s004.pdf]

Appendix S4. Deoxygenation methodology of sickle RBCs

Sickling kinetics were observed in a microfluidic device comprising a free-standing gas-permeable polydimethylsiloxane (PDMS) membrane (150m thick) within a dual-layer microchannel construction. Device design principles are described in detail in [1]. In brief, the dual-layer device comprises a "*flow microchannel*" in which there is flow of RBC suspension; and a "*control microchannel*" in which the desired gas mixture can be delivered and its partial pressure regulated. Within the "*control microchannel*" pressurized gas - of predefined oxygen concentration - is delivered via a high precision pneumatic pump and diffuses channel through the PDMS membrane to the "*flow microchannel*". The two oxygen states mentioned in the text are the following: A. ***Oxy state*** i.e., O_2 concentration 20% [gas mixture of 5% $CO_2, 20\%$ O_2 with N_2 balance] and B. ***DeOxy state*** i.e., O_2 concentration: 2% [gas mixture of 5% $CO_2, 2\%$ O_2 with N_2 balance].

References

1. Du E, Diez-Silva M, Kato GJ, Dao M, Suresh S. Kinetics of sickle cell biorheology and implications for painful vasoocclusive crisis. Proceedings of the National Academy of Sciences. 2015;112(5):1422–1427.
